# Abstract Book - 4^th^ B Chromosome Conference: Botucatu, Brazil. 20 - 23 July 2019

**DOI:** 10.1186/s12919-019-0165-x

**Published:** 2019-06-17

## I-01 Welcome to the 4^th^ B Chromosome Conference

### Cesar Martins (Chair: 4th B Chromosome Conference)

#### Institute of Biosciences at Botucatu, São Paulo State University (UNESP), Botucatu, SP, Brazil

**Correspondence:** Cesar Martins (cesar.martins@unesp.br)

**Organization**

Institute of Biosciences at Botucatu, São Paulo State University (UNESP)

**Support**

International Chromosome and Genome Society

Brazilian Genetics Society

**Funding Support**

The conference and this publication was funded by Fundação de Amparo à Pesquisa do Estado de São Paulo - Brazil (grant number 2018/25753-5); Coordenação de Aperfeiçoamento de Pessoal de Nível Superior - Brazil (grant number 88887.289479/2018-00); Conselho Nacional de Desenvolvimento Científico e Tecnológico - Brazil (grant number 403525/2018­7)

**Organizing Committee**

Cesar Martins (Chair), Brazil

Andreas Houben, Germany

Patrick Ferree, USA

Vladimir Trifonov, Russia

**Local Organizer Committee and Contacts**

Cesar Martins (Chair)

Adriane Wasko

Adauto Cardoso

Camila Moreira

Érica Ramos

Jordana Oliveira

Rafael Nakajima

Email: 4bcc.ibb@unesp.br

Phone: 55(14)38800462

Conference website: https://www3.ibb.unesp.br/4bcc/

Facebook: https://www.facebook.com/4thbcc/

**Advisory Board**

Adriane Wasko, Brazil

Andreas Houben, Germany

Cesar Martins, Brazil

Patrick Ferree, USA

Vladimir Trifonov, Russia

**Venue**

IBB Conference Center, Institute of Biosciences at Botucatu, São Paulo State University (UNESP), Botucatu, Brazil

B chromosomes (Bs) are enigmatic accessory elements to the regular chromosome set (A) and, since their discovery at the beginning of 20^th^ century, Bs have ranked among the main topics of chromosome biology. B chromosome science has advanced from classical (conducted during most of the 20th century) and molecular cytogenetics (conducted from 1990-2010) to genomics and bioinformatics approaches (conducted during the last few years). Recent advances in next generation sequencing (NGS) technologies and high-throughput molecular biology protocols have led to B chromosomes becoming the subject of massive data analysis, thus enabling an investigation of structural and functional issues at a level that was previously never considered possible. Therefore, the 4^th^ BCC in Brazil is an excellent opportunity to bring exciting B-chromosome related topics and new technologies together for discussion under the view of the most outstanding chromosome biologists. Furthermore, this meeting will, for the first time, promote an educational activity focused on science and biology teachers in order to highlight methodologies and practical approaches that can be used in chromosome teaching/learning.

The importance of B chromosomes is illustrated by the series of conferences on this issue that have been organized during the last three decades - 1^st^, 2^nd^ and 3^rd^ B Chromosome Conferences - organized in 1993 (Spain), 2004 (Spain), and 2014 (Germany), respectively. The first B Chromosome Conference (BCC) took place in Miraflores de la Sierra (Madrid, Spain) from 21 to 25 of September 1993. The meeting had five sessions including Polymorphisms and geographic distribution, Transmission: non-Mendelian heredity, Genetic structure and organization, Phenotypic effects, and Population dynamics. Participants representing 12 countries were involved in the B chromosome meeting giving talks and presenting posters. An excellent overview of the 1^st^ BCC was presented by Beukeboom (1994) bringing to the public the major contributions of this important scientific meeting. Among the major contribution of the 1^st^ BCC, Beukeboom highlighted: (i) the statement of JPM Camacho (Granada, Spain) that proposed the moderns view of Bs as “a dispensable supernumerary chromosome that does not recombine with the A chromosomes and follows its own evolutionary pathway”; (ii) as a major direction for the B investigation, a lot of knowledge may be gained from the molecular characterization of Bs. The 2^nd^ BCC was organized in Granada, Spain, during June 26-29 of 2004 and counted with participants belonging to 15 countries. The 2^nd^ BCC had five sessions on Frequency and meiotic behavior, Transmission, Phenotypic effects, DNA composition and origin, and Evolution. The most outstanding contribution of the meeting comes from the application of fluorescence in situ hybridization to the mapping (FISH-mapping) of DNA sequences in the chromosomes. At this moment, FISH-mapping was extensively explored for comparative analysis. The 3^rd^ B Chromosome Conference was conducted on April 7- 9 of 2014 in Gatersleben, Germany. Participants coming from 11 different countries gathered to discuss the latest development in the following B chromosome research areas: Structure and evolution of animal and plant B chromosomes; B chromosome effects and genes; Population genetics of B chromosomes; Segregation behavior and drive of B chromosomes; and Novel analysis methods and application of B chromosomes. Beside posters, 22 talks were given from scientists leading the area of supernumerary chromosome research. The most highlighted issue of the 3^rd^ B edition was the exploration of high scale analysis of DNA and RNA that raises the B chromosome biology to a different level.

Therefore, the three previous editions of the B Chromosome Conference highlighted that B chromosome biology has advanced a lot under the impact of recent development of genomics and bioinformatics tolls and functional approaches. So, it is timely to consider a new edition of the B chromosome forum, considering the fast advancing of B chromosome studies in the last few years (for reviews Ahmad and Martins 2019; Houben et al. 2019). In this way, you are invited to join us in Botucatu, Brazil, in July 20-23 of 2019, for an excellent opportunity to bring exciting B-chromosome related topics and new technologies together for discussion under the view of the most outstanding chromosome biologists. The 4^th^ BCC will include 18 talks, 16 short talks, and poster presentations on diverse topics as Education in chromosome Science, Structure, composition and evolution of B chromosomes, Genes and B chromosome effects, Population genetics of B chromosomes, Segregation behavior and drive of B chromosomes, and New technologies and applications of B chromosomes.

**References**

Ahmad SF, Martins C. The modern view of B chromosomes under the impact of high scale omics analyses. Cells. 2019; 8: 156.

Beukeboom LW. Bewildering Bs: an impression of the 1st B-Chromosome Conference. Heredity. 1994; 73: 328-336.

Houben A, Jones N, Martins C, Trifonov V. Evolution, composition and regulation of supernumerary B chromosomes. Genes. 2019; 10: 161.

## S0- Key note talk - The history of B chromosomes

### Neil Jones

#### Aberystwyth University, Aberystwyth, UK

**Correspondence:** Neil Jones (neil.rnj@gmail.com)

**Background**

The history of B chromosomes is written in all the publications’ produced on this topic over the last 110 years. The first 75 years of the world literature is recorded in the book *B Chromosomes* (Jones and Rees 1982). This publication contains 1,376 references between 1906 to 1980, all of which were collected as hard copy (reprints and photocopies) with the objective of gathering the world literature up to this point in time. The lecture will briefly explain how all of these papers were found before literature searches became available. Following 1980 onwards numerous papers were collected by myself as PDF files (1054 files to date). A further valuable resource of 41 articles appeared in *B Chromosomes in the Eukaryote Genome*, edited by Juan Pedro Camacho (Camacho 2004) Progress with the history was then recorded up to the present time in numerous review manuscripts, culminating in 17 precious articles (8 articles, 9 reviews) in the Special Issue of Genes 2019: *"Evolution, Composition and Regulation of Supernumerary B Chromosomes"* (Houben et al. 2019)*.* Occurrence of Bs in major eukaryotic groups can be found in the B chrom database (http://www.bchrom.csic.es/) which deals with all the major groups of organisms from fungi to mammals. The lecture will track the history of Bs from this mass of available literature and point to the key elements of the story from its beginning in from 1906 to the present time.

**References**

Jones RN, Rees H. B Chromosomes. Academic Press: London, UK, 1982.

Camacho JP. B chromosome in the eukaryote genome. Cytogenet Genome Res. 2004; 106 (2-4):143-413.

Houben A, Jones N, Martins C, Trifonov V. Evolution, composition and regulation of supernumerary B chromosomes. Genes. 2019; 10: 161.

## Session 1 - Education in chromosome science

### S1-01 Hands on Science: obtention, visualization, and analyses of human chromosomes

#### Adriane P Wasko, Lígia SLS Mota

##### Department of Genetics, Institute of Biosciences at Botucatu, São Paulo State University (UNESP), Botucatu, SP, Brazil

**Correspondence:** Adriane P Wasko (a.wasko@unesp.br)

**Background**

Students´ positively involvement in learning approaches, especially in high school, is a huge challenge. This fact can be related to several circumstances, as no innovative and practical classes, inadequate school infrastructure, excessive number of students at the same class, and even teachers downgrading. As Science and its Technologies have a high importance to anybody´s critical instruction, they should be appropriately transmitted to all educational grades. Moreover, science education and divulgation activities should not be assumed only by basic school, but also by universities. Therefore, the present work - that is related to a practical course on Cytogenetic issues - intend to use the facilities of the São Paulo State University in order to allow basic school teachers to take part of some lab experiments and analyses.

**Materials and Methods**

Hands on science activities will be directed to teachers in order to promote the capacity to generate theoretical-practical activities in schools based on the High School curricular contents of Genetics, especially Cytogenetic issues. Themes as phenotype and genotype, alleles, autosomal and sex linked inheritances, karyotype, structural and numerical chromosome alterations, and nucleic acids structure will be explored throughout educational kits, models, games, and group dynamics. Moreover, practical activities will be performed in order to obtain, visualize, and analyze mitotic human chromosomes throughout blood cell cultures.

**Results**

The participants will have the opportunity to elaborate an educational material (chromosome slides and karyotypes) to use in further classroom activities. We intend to endorse the training of Biology and Science teachers as also encourage their interest in research in the field of Genetics.

**Conclusions**

The proposed actions subsidize not only science popularization activities, but also the university consolidation as a teaching and research institution and resolution of social and inequality problems, and are in conformity to national politics for basic education, in order to transpose Science and Technology data to basic school.

**Acknowledgements**

This research was funded by São Paulo Research Foundation - FAPESP, grant number

2015/16661-1.

## P1-01 Karyotyping teaching kit

### Laura C Benvindo^1^, Mariana A Dias^1^, Maurício JB Pereira^1^, Pedro Henrique Castro^1^, Gabriela M Guimarães^1^, Maria EF Barros^1^, Thais Inácio^1^, Barbara CS Fernandes^1^, Fawaz A Jammal Filho^1^, Marielly de Campos^2^, Lígia SLS Mota^2^, Adriane P Wasko^2^

#### ^1^Colégio Embraer “Casimiro Montenegro Filho”, Botucatu, SP, Brazil; ^2^Department of Genetics, Institute of Biosciences at Botucatu, São Paulo State University (UNESP), Botucatu, SP, Brazil

**Correspondence:** Adriane P Wasko (a.wasko@unesp.br)

**Background**

Genetics is an essential science nowadays and, for this reason, it’s a matter of notorious importance teaching it appropriately to achieve the best levels of comprehension. However, the current situation in Brazilian’s education is something extremely precarious, especially in public institutions. The most worrying factor is when undergraduates start college, with a huge lack of knowledge in certain curricular components. In the Biological Sciences area, due the complexity of the subject, there is a great understanding problem in Genetics topics by the students. The objective of this study was to elaborate a new educational and interactive material which does not require many resources in order to teach Genetics, especially cytogenetics and several chromosomal alterations, to high school students from public and private schools.

**Materials and Methods**

The prototyping of a pedagogical material to teach Genetics was developed by high school students from Colégio Embraer “Casimiro Montenegro Filho”, in partnership with the Institute of Biosciences of UNESP, Botucatu Campus. Forty seven magnetized plastic pieces representing chromosomes that constitute the human karyotype (female or male); five extra pieces representing numerical changes (as Down, Edwards, Patau, Turner, and Klinefelter syndromes); two larger pieces with modular parts, each representing structural alterations in the chromosomes (as deletion, duplication, inversion, and translocation); one metal board for securing parts; one explanatory guide; and one case for transportation and storage of the product.

**Results**

Due to the complexity of the subject, we conclude that the proposed material seems to be a good choice to make Genetics learning for high school students more interactive, interesting, dynamic, and easy to understand. A pilot class was held with professors of Biology and 3rd grade students from the Embraer College, in which the interactive material was applied so that it could be evaluated and possible doubts and suggestions could be raised in order to improve it. However, it is still necessary to use the interactive material in test lessons to verify the effectiveness of the learning process and/or to improve it.

**Conclusions**

It should be emphasized that there are no similar educational materials in the national or international market and that the next steps in this work refer to the production of an educational kit in partnership with a Brazilian company and subsequent referral of the INPI (National Institute of Industrial Property) letter to obtaining a patent.

**Acknowledgements**

This research was funded by São Paulo Research Foundation - FAPESP, grant number

2015/16661-1.

## Session 2 - Structure, composition and evolution of B chromosomes

### S2-01 Recent progress in understanding B chromosome evolution

#### Robert Trivers

##### Chapman University, Orange, CA, USA

**Correspondence:** Robert Trivers (triversr@gmail.com)

B chromosomes were last reviewed in 2006 as part of the larger literature on Selfish Genetic Elements (Burt and Trivers 2006). Work has exploded since then. We now know of additional cases of B’s evolving to become neutral - in one case by developing drag in females to counterbalance drive in males. Is this an organism fighting back against its driving B? We know the location of active genes on B’s in some species and their putative function. We know of newly evolved Bs that are an amalgam of repetitive DNA, ribosomal and others. We know that B’s sometimes act as sinks for retro-transposons but differ in whether this reflects absence of cost to the retro or benefit to the Bs. We are just beginning with the genomics of Bs. I will review the topic from 2006 to 2019.

**Reference**

Burt A, Trivers R. Genes in conflict: the biology of selfish genetic elements. Belknap Press of Harvard University Press, Cambridge, 2006.

### S2-02 The origin and evolution of apomixis-related chromosomes in *Boechera* (Brassicaceae)

#### Terezie Mandáková^1^, Michael D Windham^2^, Thomas Mitchell-Olds^2^, Martin A Lysak^1^

##### ^1^CEITEC - Central European Institute of Technology, Masaryk University, Brno, Czech Republic; ^2^Department of Biology, Duke University, Durham, NC, USA

**Correspondence:**  Terezie  Mandáková (Terezie.Mandakova@ceitec.muni.cz)

**Background**

Apomixis is rare in the mustard family. Here our aim was to investigate the genome structure and evolution of sexual and apomictic *Boechera* diploids (2n = 2x = 14, 15) and triploids (2n = 2x = 22), and to elucidate the origin of heterochromatic B-like chromosomes in apomictic accessions. To this end we used comparative chromosome painting (CCP) analysis with differentially labelled BAC contigs of *Arabidopsis thaliana* arranged according to the structure of a purported ancestral genome with eight chromosomes (Ancestral Crucifer Karyotype, ACK). First, we constructed comparative cytogenetic maps for sexual and apomictic diploids (2n = 14), and aneuploid apomictic accessions (2n = 15, 22) and reconstructed the origin of the diploid genomes from the ACK. In *Boechera*, three chromosomes retained the ancestral ACK-like structure, whereas the four remaining chromosomes originated through reciprocal translocations and inversions reshuffling the ancestral genomic blocks and reducing the number of linkage groups from 8 to 7. Two heterochromatic B-like chromosomes (*Het* and *Del*) have been described in *Boechera* apomicts. In eudiploid apomicts (2n = 14), the *Het* chromosome was identified as one of the B01 homologues comprising blocks A, C and D. In aneuploid apomicts (2n = 15, 22), the *Het* chromosome consists only of blocks A and C. The block D has become the telocentric *Del* chromosome with a centromere originated either by centric fission of *Het* or *de novo* formation. Interestingly, in some aneuploid apomictic accessions, the *Het* was reshuffled by a pericentric inversion, additionally followed by a reciprocal translocation in other accessions. The origin and structure of *Het* and *Del* chromosomes are important for further understanding genome evolution in apomictic *Boechera* plants.

**Acknowledgements**

This work was supported by Ministry of Education, Youth and Sports of the Czech Republic within the programme INTERACTION (LTAUSA17002).

### S2-03 Origin and evolution of mammalian B chromosomes

#### Vladimir A Trifonov, Alexey I Makunin, Dmitry Yu Prokopov, Ilya G Kichigin, Anna S Druzhkova, Svetlana S Romanenko, Kristina O Petrova, Alexander S Graphodatsky

##### Institute of Molecular and Cellular Biology Siberian Branch of the Russian Academy of Sciences, Novosibirsk 630090, Russia

**Correspondence:** Vladimir A Trifonov (vlad@mcb.nsc.ru)

**Background**

B chromosomes (Bs) have been described in around 70 species of mammals (50 of these species are rodents). Taking the advantage of sequenced reference genomes of related species we sequenced isolated B chromosomes of carnivores (red fox and raccoon dog), ruminants (Siberian roe deer and brown brocket deer) and rodents (collared lemming and two species of *Apodemus*), and demonstrated that Bs are enriched in segmental duplications derived from different genomic regions. Our data further confirm that these duplicated copies are subjected to pseudogenization and repeat accumulation. We demonstrate independent origin of B chromosomes in different species and the phenomenon of parallel re-use of the same genomic regions in different lineages. Comparison of B-specific gene content of canids, ruminants, and rodents strongly indicates enrichment with cell-cycle and development related functions. The study of repetitive DNA repertoire shows the enrichment of B chromosomes in particular classes of elements. Mammalian Bs content shows many common features to this of other vertebrates, which may be regarded as parallel evolution.

**Acknowledgements**

This research was funded by RSF grant number 19-14-00034.

### S2-04 The history of *Cestrum* (Solanaceae) B-chromosomes reveals intense rearrangements in the repetitive DNA families, with conservation of chromosomes morphology and size

#### Thaissa B de Souza, Marcos L Gaeta, André L L Vanzela

##### Department of General Biology, Center for Biological Sciences, State University of Londrina, Londrina, PR, Brazil

**Correspondence:** André Vanzela (andrevanzela@uel.br)

**Background**

About 4% of angiosperms have B chromosomes. These are recognized due to their non-Mendelian inheritance, univalent behavior on meiosis and accumulation of repetitive DNA families. Despite efforts to decipher the B chromosome origin and development, aspects of their genetic composition, function and evolution remain unresolved in several plant groups. At least for Solanaceae, the Bs they appear frequently in species with large genomes, like those of *Cestrum* with 2n=16 large chromosomes with up to 12 μm each and 2C=~20 pg. Seven species carry Bs, with one-third the size of the largest A chromosomes. The Bs of *Cestrum* vary on the repetitive DNA content and distribution, like rDNA, transposable elements and SSR. Several of these sequences are shared with A chromosomes of different species.

**Materials and Methods**

The results of about ten years of cytogenetic studies with B chromosomes of *Cestrum*, including meiosis, mitosis and chromosome banding, as well as FISH with repetitive DNA probes and immunostaining, are presented here. Data available of different sources make very intriguing the origin of these Bs, because *Cestrum* is a small genus, that contains several species carrying different numbers of B.

**Results**

Solanales has about 40 species with B-chromosomes, and 30 of them in Solanaceae. *Cestrum* has seven species with Bs, and all of them are short submetacentric with a third of the size of A chromosomes. Despite this similarity, a great diversity of repetitive families has been detected in different species. Meiotic studies evidenced loss of Bs in pseudomicoresporocytes, as well as divergences in the H3 phosphorylation in relation to A chromosomes throughout the meiosis. The Bs have accumulated AT-rich minisatellites, CMA^+^/DAPI^+^ bands, Gypsy LTR-RTs and 35S and 5S rDNA in a differential manner.

**Conclusions**

Repetitive DNA families may insert, spread, and amplify independently in the Bs along evolutionary history. Differences in the order of the B chromosomes repetitive sequences, the almost total exclusion of some sequences, or intense amplification, make obscures the interpretation on the origin of these chromosomes in *Cestrum*. Karyotypes of *Cestrum* differ a lot on the repetitive families distribution, but they maintain relatively the A chromosomes sizes and shapes. Apparently, the Bs are also regulated in the same way. Sequencing of plants containing 0, 1 and 2 B chromosomes will done, in order to decipher the B driving control, functional genes, and new details on the repetitive sequences roles acting on the evolutionary history of Bs.

**Acknowledgements**

This research was funded by Paraná Araucária Foundation - FA, and National Counsel of Technological and Scientific Development - CNPq.

### S2-05 Holocentric B chromosomes in animals

#### Diogo C Cabral-de-Mello

##### Department of Biology, Institute of Biosciences at Rio Claro, São Paulo State University (UNESP), Rio Claro, SP, Brazil

**Correspondence:** Diogo C Cabral-de-Mello (cabral.mello@unesp.br)

**Background**

Supernumerary B chromosomes were described for the first time in one Hemiptera insect more than one century ago. Although this first observation was done in a species with holocentric chromosomes, studies about B chromosome biology were done mainly in species with monocentric chromosomes. In this way, there are many unanswered questions about holocentric B chromosomes.

**Materials and Methods**

I revisited the literature about B chromosomes in animals with holocentric chromosomes. Moreover the B chromosomes of the hemipteran *Aetalion reticulatum* were studied in detail for understanding the population dynamics, behavior, drive, composition of repetitive DNAs and putative origin. For this I combined classical and molecular cytogenetic methods and computational biology from sequenced genomes.

**Results**

Bs were reported only in a few animal groups with holocentric chromosomes, like Hemiptera and Lepidoptera, representing about 7.9% of the total animals harboring B chromosomes. Studies described the occurrence of B chromosomes and behavior with no details about composition. Among 300 animals of *A. reticulatum,* two variants of B chromosomes occurred. B1 is similar in size to X chromosome and occurred in constant number, but B2, that is smaller in comparison to B1, varied in number per cell at intra- and inter-follicular levels. The repitome of +B and 0B genomes revealed similar abundance of transposable elements (TEs), but accumulation of satellite DNAs (satDNAs) was noticed in the +B samples. B1 and B2 chromosomes were different for satDNAs composition and some satDNAs were extremely amplified (up to 17,000x). Concerning behavior during spermatogenesis, in anaphase, it was noticed occurrence of bridges, with B chromosomes rarely forming them. Abnormal spermatids or micronucleus were not observed.

**Conclusions**

Although holocentric B chromosomes were neglected for a long time they could bring interesting insights about B chromosome biology. The B chromosome of *A. reticulatum* is the first detailed study in a holocentric species revealing similar patterns like described in monocentric chromosomes, but with some particularities. Results point for independent and intraspecific origin of B chromosomes from autosomes, followed by accumulation of satDNAs. The differential behavior observed during spermatogenesis for two B variants could influence the population dynamics. The frequent occurrence of anaphase bridges leads to the formation of chromosome fragments. Due to holocentric nature, the chromosome fragments are maintained in the cells, offering the initial source for potential origin of new B chromosomes. Moreover the holocentric nature could also influence the maintaining of supernumerary chromosomes in genomes.

**Acknowledgements**

This research was funded by São Paulo Research Foundation - FAPESP, grant 2015/16661-1.

### S2-06 On the origin of the *Drosophila* Y chromosome

#### Antonio Bernardo Carvalho_,_ Eduardo Dupim, Thyago Vanderlinde, Fabiana Uno, Guilherme Dias, João Ricchio, Felipe Vigoder, Victor Seixas, Marcos Torres

##### Department of Genetics, Institute of Biology, Federal University of Rio de Janeiro, RJ, Brazil

**Correspondence:** Antonio B Carvalho (bernardo1963@gmail.com)

**Background**

Y chromosomes are widely believed to evolve from a normal autosome through a process of massive gene loss; the other homolog became the X chromosome. The process is initiated when one autosome acquires a strong male-determining gene, becoming a proto-Y; its homolog became the proto-X. This canonical pathway is best illustrated and supported by mammalian Y chromosomes, which contain very few genes, most of them shared with the X. However, we found no sign of X-Y homology in *Drosophila*; instead, the majority of *Drosophila* Y-linked genes are recent acquisitions from autosomes. We also found that gene gains are more frequent than gene losses in the *Drosophila* Y. Finally, *Drosophila* Y chromosomes lack a sex-determining gene. Hence it is clear that the *Drosophila* Y fits poorly on the canonical model of Y evolution, which brings the question of its origin. As suggested by Hackstein and co-workers (Hackstein et al. 1996), a possible scenario is that the *Drosophila* Y originated from a B-chromosome. In order to investigate this hypothesis we are dating the origin of the *Drosophila* Y chromosome.

**Materials and Methods**

In order to date the origin of the *Drosophila* Y we sequenced progressively more distant genera, searching for shared Y-linked genes that indicate a shared ancestry. We used Illumina sequencing and a highly efficient method for the identification of Y-linked genes.

**Results**

We found that the *Drosophila* Y chromosome shared Y-linked genes with most Drosophila species (we tested 400 *Drosophila* species), *Scaptodrosophila*, *Chymomyza* and *Colocasiomyia*. All these genera belong to the Drosophilinae subfamily of the Drosophilidae family; the origin of the *Drosophila* a Y must predate the divergence of these species. The sign of shared ancestry became unclear when we examined the Steganinae subfamily, and completely disappear outside the Drosophilidae family (*e.g.*, in the Ephydridae family).

**Conclusions**

The ancestral *Drosophila* Y chromosome seem to had originated before the divergence of Drosophilinae genera, and after the split between Drosophilidae and Ephydridae. We are sequencing species in this taxonomic range, in order to date (with the molecular clock) the origin of the *Drosophila* Y. We are also sequencing with PacBio some of these species, in order to search for clues of the origin of the Y *Drosophila* chromosome.

**Acknowledgements**

This work was supported by grants to ABC from the Wellcome Trust (207486/Z/17/Z), CAPES, CNPq and FAPERJ.

**Reference**

Hackstein JHP, Hochstenbach R, Hauschteck-Jungen E, Beukeboom LW. Is the Y chromosome of *Drosophila* an evolved supernumerary chromosome? BioEssays. 1996; 18: 317-323.

## O2-01 Cichlid fish in the B chromosome world

### Cesar Martins

#### Department of Morphology, Institute of Biosciences at Botucatu, São Paulo State University (UNESP), Botucatu, SP, Brazil

**Correspondence:** Cesar Martins (cesar.martins@unesp.br)

**Background**

B chromosomes (Bs), a type of supernumerary chromosomes, are extra karyotypic units in addition to A chromosomes (the normal chromosomes, autosomes and sex chromosomes) and found in all major eukaryotic taxa. Bs are uniquely characterized due to their non-Mendelian inheritance, and represent an example of the genomic conflict. Over the decades, their genetic composition, function and evolution have remained an unresolved query, although a few successful attempts have been made to address these phenomena. A classical concept is that Bs are selfish and abundant with DNA repeats and transposons and, in most cases, they do not carry any function. Among the extensively investigated species and taxa on the B chromosome subject, cichlid fishes have emerged as an interesting model and have contributed to unreview the complex B chromosome biology.

**Materials and Methods**

Here we report a survey on the current state of art of B chromosome investigation in cichlid fishes. Besides the description of a list of species presenting Bs, we also report the recent advances over gene and sequences hunting on Bs and their impact on the current concept of B chromosomes.

**Results**

B chromosomes were first identified in eight species from South America (*Crenicichla niederleinii*, *C. reticulate, C. lepidota*, *Gymnogeophagus balzanii, Geophagus brasiliensis*, *Cichlasoma paranaensis*, *Cichla monoculus*, *Cichla* sp.) and lately they have been identified in several species from lakes Victoria and Malawi in East Africa. Bs have been found in fourteen species from Lake Victoria (*Astatotilapia latifasciata*, *Lithocromis rubripinnis, L. rufus, Haplochromis plagiodon, H. pyrrhocephalus, H. tanaos, Haplochromis* sp*. “*purpleyellow*”, Pundamilia pundamilia, Neochromis greenwoodi, N. rufocaudalis, Haplochromis* sp*. “*Matumbi Hunter*”, Haplochromis fisheri* and *Paralabidochromis chilotes*) and seven species from Lake Malawi (*Metriaclima lombardoi, M. zebra* “Boadzulu”, *M. zebra* “Nkhata Bay”, *M. greshakei, M. mbenji, Labeotropheus trewavasae*, and *Melanochromis auratus*). In Lake Victoria Bs occur in males and females, except in one species (*L. rubripinnis*) that Bs occurs only in female. On the other hand, in all Lake Malawi species Bs were exclusively found in females, although some females do not present Bs. The Bs of cichlids were also subject of high scale DNA and RNA analysis and epigenetics and a list of B chromosome genes and functional sequences has been generated. Our results have shown that the B chromosome is enriched with genes, relics of genes, transposable elements and sequences transcribing for many significant biological functions.

**Conclusions**

Diverse potentially functional sequences have been described in the B chromosome of cichlids and could influence important biological characteristics of species. Furthermore, the B chromosome presence seems to affect transcription and epigenetic modifications of the whole genome. One of the most intriguing characteristics of Bs in cichlids is their genic content related to cell cycle and chromosome structure, and their influence over sex rates. Although the association of B chromosomes and sex in cichlids is still enigmatic, we could hypothesize that the B chromosome bias for females could favor their drive during female meiosis.

**Acknowledgements**

This research was funded by São Paulo Research Foundation (FAPESP) - grant number 2015/16661-1, and National Counsel of Technological and Scientific Development (CNPq) - grant number 305321/2015-3.

## O2-02 Do B chromosomes survive speciation events?

### Ana Beatriz S M Ferretti^1^, Diogo Milani^1^, Vilma Loreto^2^, Diogo C Cabral-de-Mello^1^

#### ^1^Department of Biology, Institute of Biosciences at Rio Claro, São Paulo State University (UNESP), Rio Claro, SP, Brazil; ^2^Department of Genetics, Center of Biology Sciences, Pernambuco Federal University (UFPE), Recife, PE, Brazil

**Correspondence:**  Ana  Beatriz  S  M Ferretti  (anabeatrizferretti@gmail.com)

**Background**

One low explored aspect and opened question about B chromosomes evolution is their possible origin in sister species prior to speciation events. The Romaleidae grasshopper *Xyleus discoideus angulatus* has a relatively well-characterized B chromosome and recently our group found one B chromosome in *X. d. discoideus*, a sister species of *X. d. angulatus*. In this work we studied the satellitome of the two *Xyleus discoideus* subspecies to try to elucidate the composition and ascertain if the B chromosomes were originated before speciation events.

**Materials and Methods**

We sequenced by Illumina Hiseq two genomes of *X.d.angulatus* and *X.d.discoideus* both 0B and +1B. Using SatMiner pipline we searched for satDNAs in the four genomes and performed a landscape analyses to verify sharing of satDNAs between species and B chromosomes and differences in proportion of each satDNA. We also collect individuals of both species for phylogenetic analyses using COI (Cytochrome Oxidase).

**Results**

Phylogenetic tree of COI shows that *X.d.discoideus* and *X.d.angulatus* form separated groups. We found 34 satDNA in *X.d.discoideus* and 32 satDNA in *X.d.angulatus*, those represents, approximately, 3.02% and 4.04% of the genomes 0B, respectively. From those satDNAs, 32 are present in the both subspecies, whereas two are specific of *X.d.angulatus* and three of *X.d.discoideus*. Both +1B genomes showed an increased amount of satDNA: *X.d.angulatus* 39% and *X.d.discoideus* 13% more than 0B. These repeats were generally amplified on B chromosome. No B specific satDNA were observed. However, we noticed the presence of two satDNAs specific of one subspecies (on 0B and +B genomes) shared exclusively with the genome +B of the other subspecies. We also observed that among enriched satDNAs on +1B genomes, five of them are shared between the both sister species.

**Conclusions**

Our data suggest the origin of the B chromosomes in both subspecies prior to speciation events. In this way after speciation the B chromosomes were maintained and accumulated some specific variation. This hypothesis is supported by: (i) sharing of amplified satDNAs in the +B genomes of both subspecies; (ii) presence of two satDNAs specific of one subspecies occurring exclusively in the +1B genome of the other subspecies, revealing similarity between B content and genome of different subspecies. The chromosomal mapping of the satDNAs will help us to shed light on this hypothesis and in the understanding of specific distribution of the repeats in the A and B chromosomes.

**Acknowledgements**

FAPESP-2015/16661-1, CAPES and CNPq funded this research.

## O2-03 Unusual low abundance of repetitive DNAs in the supernumerary chromosome of *Abracris flavolineata* revealed by genome and chromosomal analysis

### Diogo Milani^1^, Francisco J Ruiz-Ruano^2^, Juan Pedro M Camacho^3^, Diogo C Cabral-de-Mello^1^

#### ^1^Department of Biology, Institute of Biosciences at Rio Claro, São Paulo State University (UNESP), Rio Claro, SP, Brazil; ^2^Department of Ecology and Genetics, Evolutionary Biology Centre Norbyvägen, Uppsala University, Uppsala, Sweden; ^3^Department of Genetics, University of Granada, Granada, Spain

**Correspondence:** Diogo Milani (azafta@gmail.com)

**Background**

B chromosomes were first reported in 1906, but their genetical content is now beginning to be known in detail. They most likely originated from A chromosomes and, in most cases, are heterochromatic and composed of high amounts of repetitive DNA sequences which, logically, play an important role in B differentiation and evolution. One of the few exceptions is the B chromosome of the grasshopper *Abracris flavolineata*, as all previous studies suggest its euchromatic nature.

**Materials and Methods**

Illumina high-throughput sequencing and computational analyses were performed to ascertain B chromosome DNA composition in *A. flavolineata*. Sequencing of genomic DNA was performed in seven individuals: three 0B, two 1B and two 2B. Repetitive DNAs were searched for by bioinformatic approaches and mapped on chromosomes through Fluorescent *in situ* Hybridization (FISH).

**Results**

In the 0B libraries, we found 53 satellite DNA (satDNA) families (representing about 5.6% of the genome), 138 families of different classes of transposable elements (TE) (31%), and several multigene families (0.45%). Remarkably, average abundance of repetitive DNA content was similar in 0B and B-carrying genomes, but showed high variation between individuals. In fact, only Afsat52 showed much higher abundance in +B genomes, reaching almost 15-fold higher abundance in comparison to 0B genomes. Likewise, only four TEs showed higher abundance in +B genomes. Kimura divergence indicated less variation for several repeats showing higher abundance in +B genomes, suggesting recent amplification in the B chromosome. FISH mapping showed hybridization signals on the B chromosome for 11 satDNA families, in some cases being spread and in others showing conspicuous bands mainly located close to centromeric and telomeric regions of A and B chromosomes. Only the Afsat46 family shared the FISH pattern in the B chromosome with a single A chromosome, supporting the possible origin of the B chromosome from the longest autosome. Moreover, the four selected TEs showed spread FISH signals on both B chromosome arms and euchromatic regions of A chromosomes.

**Conclusions**

These results are consistent with B chromosome origin from the longest autosome via isochromosome formation. Moreover, unlike most B chromosomes, the large and euchromatic B of *A. flavolineata* is not heavily loaded of repetitive DNA, in resemblance to euchromatin in the A complement.

**Acknowledgements**

CAPES, CNPq and FAPESP-2015/16661-1 funded this research.

## O2-04 From bacterium to B chromosome: Analysis of a B chromosome in the Mediterranean flour moth, *Ephestia kuehniella,* revealed an unusual case of horizontal gene transfer

### Anna Voleníková^1,2^, Martina Dalíková^1,2^, Sander Visser^1,2^ and František Marec^1^

#### ^1^Institute of Entomology, Biology Centre of the Czech Academy of Sciences, České Budějovice, Czechia; ^2^Faculty of Science, University of South Bohemia in České Budějovice, České Budějovice, Czechia

**Correspondence:** Anna Voleníková (anna.volenikova@gmail.com); František Marec (marec@entu.cas.cz)

**Background**

Moths and butterflies (Lepidoptera) with more than 180,000 species described worldwide represent one of the largest animal orders. Yet, the presence of B chromosomes has been reported in only 25 cases and no detailed studies have been published. Here we present a thorough analysis of a B chromosome in the Mediterranean flour moth *Ephestia kuehniella*, which turned out to be more than just the first survey in this field.

**Materials and Methods**

To analyse the nature and composition of the B chromosome in *E. kuehniella*, we used methods of molecular cytogenetics and genomics. First, we performed a basic cytogenetic examination of the karyotype, followed by a series of *in situ* hybridizations with probes derived from the major repetitive regions known in Lepidoptera. However, none of the probes hybridized to the B chromosome, so we used next generation sequencing (NGS) to reveal its content. Genomes of *E. kuehniella* specimens with and without B chromosomes were sequenced at low coverage using Illumina platform, and the resulting datasets were compared and processed with the RepeatExplorer pipeline.

**Results**

Hybridization experiments showed that the B chromosome of *E. kuehniella* does not contain detectable amount of DNA repeats originating from the W chromosome, rDNA gene clusters or telomeric sequences. Surprisingly, NGS data analysis identified sequences from *Wolbachia*, an endosymbiotic bacterium present in many arthropods, as the primary source of genetic material present on the B chromosome. These results were confirmed by *in situ* hybridization, demonstrating incorporation of *Wolbachia* DNA into the host genome through the B chromosome.

**Conclusions**

Our B chromosome research in *E. kuehniella* provides evidence of a unique case of horizontal gene transfer between endosymbiotic bacteria and a B chromosome of lepidopteran host. Moreover, recent results suggest that the W chromosome in Lepidoptera might have evolved by ancient adoption(s) of a supernumerary chromosome. It has also been proposed that domestication of sequences derived from a parasite or an endosymbiont could have potentially played a role in this process. Our results thus offer an interesting connection between these hypotheses regarding the origin of the W chromosome.

**Acknowledgements**

This research was funded by Czech Science Foundation (GACR), grant numbers 14-22765S and 17-13713S, and Student Grant Agency (SGA) of the Faculty of Science, University of South Bohemia in České Budějovice, Czech Republic. A.V. acknowledges support from the travel fund of IBERA (Incorporation of the Biology centre CAS into the European Research Area) project number CZ.02.2.69/0.0/0.0/16_028/0006247.

## O2-05 What is the repeat composition of holocentric B chromosomes? The case of *Aetalion reticulatum*

### Vanessa B Bardella, Diogo Milani, Diogo C Cabral-de-Mello

#### Department of Biology, Institute of Biosciences at Rio Claro, São Paulo State University (UNESP), Rio Claro, SP, Brazil

**Correspondence:** Vanessa B Bardella (vbbardella@gmail.com)

**Background**

B chromosomes are largely composed of repetitive DNA elements. In some species, these sequences could help in the understanding of B chromosome origin and evolution. However, knowledge about B chromosome repeats composition is predominant in monocentric chromosomes. In this way, the analysis of B chromosome from *Aetalion reticulatum* (Hemiptera), which presents holocentric chromosomes, allows the comparison of composition and evolutionary patterns between monocentric and holocentric B chromosomes, bringing new general ideas about B chromosome biology.

**Materials and Methods**

We collected 300 insect males from UNESP (Rio Claro-SP, Brazil). Low-coverage sequencing was performed on one male without B chromosome (0B) and one male with B chromosome (+B, type I). The output data were used in computational analysis (SatMiner and RepeatExplorer) to prospect the repetitive DNAs, as well as the abundance comparison of the sequences found between genome +B and 0B. Chromosome location through Fluorescent *in situ* Hybridization (FISH) was performed for the most abundant repetitive DNAs in +B genome.

**Results**

Repitome analysis revealed 50 satellite DNA (satDNA) families and 93 transposable elements (TEs). For TEs no difference in abundance was observed in genomes 0B and +B, while for satDNAs the abundance in genome +B was about 1% more in comparison to 0B genome. For the most abundant TEs (14) FISH mapping did not reveal clustered signals. On the other hand, the most abundant satellite DNA (nine) were detected on chromosome B. Interestingly, based on satDNA composition it was detected occurrence of two types of B chromosome. B chromosome type I shared with the complement A four satellite DNAs (Arsat 3, Arsat 5, Arsat 10, Arsat 11), with Arsat 49 and Arsat 50 enriched exclusively on this element. On the other hand, the B chromosome type II shared with the complement A two satellites DNA (Arsat 11 and Arsat 46), with the Arsat 20 located exclusively on B complement.

**Conclusions**

*Aetalion reticulatum* B chromosomes revealed exclusive enrichment of satDNAs, despite the great diversity and quantity of TE in 0B genome. Sharing of satDNAs between A and B chromosomes reveals intraspecific origin, followed by massive amplification of some satDNA families on B chromosomes. Due to the absence of satDNAs exclusive of X chromosome we suggest that both types of Bs were originated from autosomes. The low similarity satDNA composition between the two types of B chromosomes indicate independent origin.

**Acknowledgements**

This research was funded by São Paulo Research Foundation - FAPESP, grant numbers 2018/21772-5 and 2015/16661-1.

## O2-06 A big puzzle named B chromosomes in *Partamona* bees

### Vander C Tosta^1^, Marco A Del Lama^2^, Lucio A O Campos^3^

#### ^1^Department of Agricultural and Biological Sciences, Espirito Santo Federal University (UFES), São Mateus, ES, Brazil; ^2^Department of Genetics and Evolution, Sao Carlos Federal University (UFSCAR), São Carlos, SP, Brazil; ^3^Department of General Biology, Viçosa Federal University (UFV), Viçosa, MG, Brazil

**Correspondence:** Vander Tosta (vander.tosta@ufes.br)

**Background**

B chromosomes are "extra chromosomes” to the normal complement that have a distinct pattern of inheritance of the usual (Mendelian) between individuals that show them. In *Partamona* species were registered fourteen types of B chromosomes in *Partamona helleri*, two types of B chromosomes in *Partamona rustica* and three types of B chromosomes in *Partamona cupira*. Furthermore, there are evidences that *Partamona criptica*, *Partamona seridoenseis*, *Partamona chapadicola* and *Partamona gregaria* may have B chromosomes too. The scenario that emerges from analysis of B chromosomes in *Partamona* species is the scenario of a big puzzle.

**Case Report**

We report a retrospective of twenty-five years of study of B chromosomes in *Partamona* species. These studies started with discovery of B chromosomes in the specie *Partamona helleri* and moved on to the evidences of B chromosomes in other species of the genus. Present studies with cytogenetic and molecular genetics data (some unpublished) gave us clues on how to unravel the mysteries about the origin and evolution of these B chromosomes. We demonstrate using SCAR (Sequence characterized amplified region) molecular markers associated with B chromosomes that the origin of the B chromosomes is basal in the phylogeny of the genus. Assuming the association between the SCAR marker and the presence of B chromosomes in *Partamona* species, origin by introgression is the hypothesis that better explains the evolution of these chromosomes in the species analyzed up to the present. Although this is the more plausible hypothesis for the origin and evolution of B chromosomes in *Partamona*, some results are difficult to explain on the basis of this assumption.

**Conclusions**

To have a better comprehension of *Partamona* B chromosomes big puzzle we propose three kinds of study: 1) The use of genomic and cytogenomic technologies (never used in *Partamona* B chromosomes studies), which would allow a more complete view of the problem; 2) A meticulous study of the transmission of *Partamona* B chromosomes intraspecifically and interspecifically; 3) A revision of the phylogeny of *Partamona*, that today is mainly based in morphological data.

**Acknowledgements**

We thank Dr. João M. F. Camargo (*in memoriam*) and Dr. Silvia R. M. Pedro (São Paulo State University - Brazil) for the taxonomic identification of *Partamona* specimens. We also thank Dr. Juan Pedro Martínez Camacho (Universidad de Granada - Spain) to helped us in the analysis.

## P2-01 Analysis of organellar DNA insertions in the B chromosome of maize

### Mohamed El-Walid, Hua Yang, James Birchler, Kathleen Newton

#### Division of Biological Sciences, University of Missouri, Columbia MO 65211 USA

**Correspondence:** Kathleen Newton (NewtonK@Missouri.edu)

Maize nuclear genomes contain insertions of both mitochondrial and chloroplast DNA – referred to as nuclear mitochondrial sequences (NUMTs) and nuclear plastid sequences (NUPTs), respectively. Using fluorescent *in situ* hybridization (FISH), we have demonstrated that the supernumerary B chromosome of maize also contains large insertions of mitochondrial DNA. The exact origin of the maize B chromosome is uncertain; however, previous research has determined its structure to be mostly heterochromatic, containing a collection of repetitive sequences. Currently, we are using a draft B-chromosome sequence produced by an international consortium to compare the B-chromosome NUMTs to the mitochondrial genotypes of maize and its relatives. Identifying the origins of the B-chromosome NUMTs may provide insight into whether they are from a recent contributor or from a more distant evolutionary source. Funding from National Science Foundation grant IOS-1444514.

## P2-02 The maize B-chromosome sequence

### Nicolas Blavet^1^, Hua Yang^2^, Handong Su^3^, Jinghua Shi^4^, Guy Kol^5^, Jonathan C. Lamb^6^, Thomas Ream^6^, Kelly Dawe^7^, Fangpu Han^3^, James A. Birchler^2^, Jan Bartoš^1^

#### ^1^Institute of Experimental Botany AS CR, Šlechtitelů 31, CZ-78371 Olomouc - Holice, Czech Republic; ^2^Division of Biological Sciences, University of Missouri, Columbia, MO 65211, USA; ^3^State Key Laboratory of Plant Cell and Chromosome Engineering, Institute of Genetics and Developmental Biology, Chinese Academy of Sciences, BeiJing, China; ^4^Bionano Genomics, San Diego, CA 92121, USA; ^5^NRGene Ltd, Ness Ziona, Israel; ^6^Bayer Crop Science, 700 Chesterfield Pkwy W, Chesterfield, MO 63017, USA; ^7^Departments of Plant Biology and Genetics, University of Georgia, Athens, GA 30602, USA

**Correspondence:** Nicolas Blavet (blavet@ueb.cas.cz)

**Background**

Hundred years ago, B chromosomes (Bs) have been discovered in maize. The particular behavior of these supernumerary chromosomes have led to the discovery of drive phenomenon and non-disjunction at the second pollen mitosis. Several studies were pointing the centromere as the key region for the non-disjunction to occur and investigations were then focusing on repetitive elements. While for decades Bs were thought to not contain any genes, recent works found the B chromosomes to be transcriptionally active.

**Materials and Methods**

In the present study, we are reporting the sequencing and assembly of the maize B chromosome. We have used a combination of technologies to achieve the present assembly: chromosome flow-sorting, Illumina sequencing, DenovoMagic assembly, BioNano optical mapping and Hi-C based chromosome-scale scaffolding. The annotations have been realized using the annotation pipeline used for the maize genome version 4, which is based on MAKER and tools dedicated to find the different families of repetitive elements.

**Results**

We estimated the size of the maize B chromosome that is about 140 Mb. The first reference assembly of this supernumerary chromosome have resulted in an 110 Mb-long pseudomolecule representing about 80% of its length. Our preliminary results of B-chromosome annotation have indicated presence of about 500 genes, while it is more than 95% composed of repetitive elements.

**Conclusions**

The sequence of the maize B chromosome is reported for the first time. It will be of great help to discover the mechanisms behind the non-disjunction of the B chromosome and the preferential fertilization of the egg cell by sperm nuclei containing Bs.

**Acknowledgments**

This project was supported by the Czech Science Agency grant 18-12338Y.

## P2-03 Repetitive DNA content of Bs, X and Y chromosomes of the rodent *Holochilus sciureus* (Cricetidae: Sigmodontinae: Oryzomyini)

### Camila N Moreira^1^, Érica Ramos^1^, Ivan R Wolf^2^, Guilherme T Valente^2^, Yatiyo Yonenaga-Yassuda^3^, Karen Ventura^3^, Cesar Martins^1^

#### ^1^Department of Morphology, Institute of Biosciences at Botucatu, São Paulo State University (UNESP), Botucatu, SP, Brazil; ^2^Department of Bioprocess and Biotechnology, Agronomic Science School, São Paulo State University (UNESP), Botucatu, SP, Brazil; ^3^Department of Genetics and Evolutive Biology, Institute of Biosciences, University of São Paulo, São Paulo, SP, Brazil

**Correspondence:** Camila N Moreira (cnmbio@usp.br)

**Background**

B chromosomes (Bs) are defined as additional dispensable chromosomes present in some individuals from some species. The use of chromosome-specific DNA libraries has allowed detailed analysis of the origin and molecular structure of Bs in different species. In rodents of tribe Oryzomyini hybridization of Bs, X, and Y chromosome probes of *Holochilus sciureus* (HSC) on 12 Oryzomyini species revealed that Bs and sex chromosomes of these species share a common heterochromatic region. This region possibly arose on the sex chromosomes of an ancestral species and spread through the action of transposable elements (TEs). However, poorly is known about the presence of TEs on the B and sex chromosomes of Oryzomyini species.

**Materials and Methods**

Probes of B1, B2, X and Y chromosomes of HSC, previously obtained by flow sorting, were subjected to sequencing using Nextera DNA Library Prep Kit in Illumina MiSeq. Obtained reads (~250 bp) were analyzed by RepeatMasker to retrieve repetitive elements. In addition, the quality of reads was checked (FastQC) and after filtering (BBDuk) the selected reads were assembled (Mira). Assemblies of B1, B2, X and Y chromosomes were aligned against each other using Mauve. Common sequences between these chromosomes were subject to BLAST search at the NCBI database.

**Results**

A total of 46920, 61400, 180166 and 28476 Illumina reads of B1, B2, X and Y chromosomes, respectively, were used in the analysis of RepeatMasker. Clusters of SINEs, LINEs, LTRs and satellite DNAs were found on B1, B2, X and Y chromosomes, moreover both B chromosomes presented rRNA sequences. tRNA and DNA.hAT.Charlie were found only in B1 and B2, respectively. The analysis of repetitive DNA associated with the alignment of B1, B2, X and Y chromosome sequences and BLAST search allowed the identification of two LTRs, Gypsy and MysTR, highly present on these chromosomes. The presence of Gypsy and MysTR on B1, B2, X and Y chromosomes of HSC suggest its association with the heterochromatic region shared among Bs and sex chromosomes of Oryzomyini species.

**Conclusions**

Previously studies reported the accumulation of MysTR in blocks or in heterochromatic regions on the genome of an Oryzomyini species, *Oryzomys palustris*. However the presence of MysTR on B chromosomes was not previously explored. Concerning Gypsy, its presence was reported on B chromosomes in the cichlid fish *Astatotilapia latifasciata*. The use of next generation sequence technology proved to be of great impact revealing elements involved in the B chromosomes composition of Oryzomyini species.

**Acknowledgements**

Research funded by São Paulo Research Foundation - FAPESP, grant numbers 2015/16661-1 and 2018/09553-6.

## P2-04 State of the art of the widely distributed B chromosomes of the tree frog *Boana albopunctata* (Anura, Hylidae)

### Juan M Ferro^1^, Kaleb P Gatto^2^, Flavia Netto^3,4^, Juan J Resquín^3,4^, William P Costa^2^, Dardo A Martí^1^, Luciana Lourenço^2^, Diego Baldo^1^

#### ^1^Laboratorio de Genética Evolutiva, Instituto de Biología Subtropical (CONICET-UNaM), Facultad de Ciencias Exactas Químicas y Naturales, Universidad Nacional de Misiones, Posadas, Misiones, Argentina; ^2^Departamento de Biologia Estrutural e Funcional, Instituto de Biologia, Universidade Estadual de Campinas (UNICAMP), Campinas, SP, Brazil; ^3^Instituto de Investigación Biológica del Paraguay, Asunción, Paraguay; ^4^Itaipu Binacional, División de Áreas Protegidas Dirección de Coordinación Ejecutiva, Ciudad del Este, Alto Paraná, Paraguay

**Correspondence:** Juan Martín Ferro (ferrojm@gmail.com)

**Background**

*Boana albopunctata* is a Neotropical frog that is widely distributed in the central and eastern region of South America. In this frog, variations in the standard chromosome number of 2n = 22 were previously described due to the presence of B chromosomes. The main goal of this work was to summarize our current knowledge about the B chromosomes of *B. albopunctata*. In order to gain information about the supernumeraries of this particular frog, and about anuran B chromosomes in general, we studied these elements from different approaches.

**Materials and Methods**

We have cytogenetically analyzed 350 specimens of *Boana albopunctata* collected in different localities of Argentina, Brazil, and Paraguay. We characterized the meiosis and mitosis of B chromosomes, describing in a preliminary way their behavior, banding patterns, and population prevalence. With the aim of evaluating the possible origin of these elements, chromosome painting was carried out with B probes obtained from microdissected chromosomes.

**Results**

Most specimens of this species had a standard karyotype with 2n = 22, and near 29% showed 1 to 3 B chromosomes. Supernumeraries were present in almost all populations with a clinal pattern of variation in their prevalence, with frequencies ranging from 5 to 50%. In the sampled geographical range (ca. 1000 km), almost all Bs were similar in morphology (metacentric), size (smaller than the smaller pair), and in the DAPI fluorochrome pattern. Chromosome painting with a B probe showed exclusive hybridization to Bs, confirming apparent homology between elements of different populations, although subtle differences in size and C-bands among Bs of particular populations were detected. During the early stages of meiosis, these elements were differentiable to standard or A chromosomes due to their pyknotic pattern, being positive in prophase I and negative in metaphase I. Moreover, these elements segregate early without dividing their sister chromatids, and without generating gross negative effects on the endophenotype of the cell carrying them.

**Conclusions**

Our results suggest the occurrence of a common morph of a B chromosome in *Boana albopunctata* that is widely distributed among populations, representing thus far the most broadly ranged B chromosome among anurans. The chromosome microdissection and painting confirmed further homology between Bs and a common origin for these elements. Likewise, the remarkable differences observed in their population prevalence, reveal our current ignorance about their biology, remaining to uncover the possible causes of that promoted this feature.

**Acknowledgments**

This study was partially funded by PICT 2015-2381, Proyecto 16Q001-TI (700-18), Universidad Nacional de Misiones, Argentina

## Session 3 - Genes and B chromosome effects

### S3-01 A B chromosome and a bacterial symbiont induce genome elimination in the jewel wasp, *Nasonia vitripennis*, through a similar chromatin mechanism

#### Haena Lee^1^, John H Werren^2^, Patrick M Ferree^1^

##### ^1^W. M. Keck Science Department of Claremont McKenna, Pitzer, and Scripps Colleges, Claremont, California, USA; ^2^Biology Department, University of Rochester, New York, USA

**Correspondence:** Patrick Ferree (PFerree@kecksci.claremont.edu)

**Background**

Genome elimination events are essential for development in a wide range of higher eukaryotes. However, two different genome parasites – an obligate intracellular bacterium known as *Wolbachia* and a B chromosome known as PSR (for Paternal Sex Ratio) – cause unprogrammed genome elimination of the sperm-inherited half of the genome in the jewel wasp, *Nasonia vitripennis*, in order to enhance parasite transmission. We previously showed that PSR causes paternal genome elimination in the wasp by disrupting three post-translational histone modifications – H3K9me2,3, H3K27me1, and H4K20me1 – during the first embryonic mitotic division. However, little is known about the chromatin basis of genome elimination caused by *Wolbachia*, and, moreover, whether these two agents cause genome elimination through common mechanisms.

**Materials and Methods**

In order to address these issues, we performed immunofluorescence confocal microscopy to visualize chromatin states in young, *Wolbachia*-affected embryos.

**Results**

We have found that *Wolbachia*, like PSR, disrupts the H3K27me1 and H4K20me1 marks, but not the H3K9me2,3 mark. Other marks, including H4ac (acetylated histone H4) and H3K27me3, were unperturbed by both agents. We also tested the rescuing effect of *Wolbachia* in the egg on the altered marks; interestingly, H3K27me1 was reversed to a normal pattern, while H4K20me1 remained abnormal.

**Conclusions and Perspectives**

Taken together, these findings suggest that disruption of H3K27me1 is a primary means by which *Wolbachia* induces paternal genome elimination, whereas H4K20me1 is secondary and insignificant in this effect. Broadly, these two different agents – *Wolbachia* and PSR – may target similar but not identical chromatin factors.

**Acknowledgements**

This work was supported by a United States National Science Foundation CAREER Award (NSF-1451839) to PMF.

### S3-02 Composition and gene expression of B chromosomes in *Astyanax* tetra fish: common origin and parasitic behavior

#### Duílio MZA Silva^1^, Francisco J Ruiz-Ruano^2^, Ricardo Utsunomia^1^, Juan Pedro M. Camacho^2^, Fausto Foresti^1^

##### ^1^Department of Morphology, Institute of Biosciences at Botucatu, São Paulo State University (UNESP), Botucatu, SP, Brazil; ^2^ Department of Genetics, Faculty of Science, University of Granada, Granada, Andalucía, Spain

**Correspondence:** Duílio MZA Silva (duiliozerbinato@gmail.com)

**Background**

B chromosomes carry redundant genetic information already present in the standard (A) chromosomes. In many cases, the presence of B chromosomes is harmful for host fitness and they are thus considered parasitic genomic elements. The 140 species of *Astyanax* make it the most diverse genus of the Characiformes order. Up to date, B chromosomes were reported in 11 species of *Astyanax*. Remarkably, the most commonly found B chromosome is a large metacentric of isochromosome nature. Shape and size similarity led some authors to suggest the common origin of B chromosomes in several *Astyanax* species, and recent results on repetitive DNA content was consistent with this hypothesis.

**Materials and Methods**

Here we analyze the protein-coding gene content of the large metacentric B chromosome in four species of *Astyanax*. We first searched for this kind of genes in *A. scabripinnis* and *A. paranae* by means of an approach recently devised, consisting in analysing the overabundance of some DNA sequences in B-carrying Illumina libraries compared with B-lacking ones, after mapping on a *de novo* transcriptome built for the same species. We then validated the results obtained by qPCR and tested the presence of the confirmed genes in *A. bockmanni* and *A. fasciatus*. In addition, we look for B-specific sequences and verify if these sequences are transcribed in 1B individuals.

**Results**

Methods used provided a list of candidate genes to be present in the B chromosome, a high number of which where then validated by qPCR. A total of 20 genes were validated in the B chromosome of *A. scabripinnis*, 19 of which were also present in the B chromosome of *A. paranae*. In addition, the B chromosome in *A. bockmanni* carried all the tested genes (13) whereas the B chromosome of *A. fasciatus* carried 11 out of the 19 tested. We detected B-specific sequences for 17 and 15 genes in *A. scabripinnis* and *A. paranae*, respectively. However, very few of these sequences were found in the transcriptome of 1B individuals.

**Conclusions**

The likelihood that these coincidences in gene content among B chromosomes in these four species would be due to chance is negligible and constitute a strong support to the common descent of these B chromosomes from a B-carrying ancestor species. The presence of the B chromosome is associated with the differential expression of many genes but, in the tissues analyzed, few B-specific sequences were present in the RNA libraries, suggesting that B chromosome genes may be mostly silenced in the stages analyzed. However, the possibility of transcription for B chromosome genes showing the same sequence as A chromosomes cannot be ruled out.

**Acknowledgements**

This research was funded by São Paulo Research Foundation – FAPESP, grant number 2017/22447-8.

## O3-01 Status of B chromosomes’ transcriptional activity in yellow-necked mouse *Apodemus flavicollis*

### Marija Rajičić^1^, Alexey I. Makunin^2^, Tanja Adnađević^1^, Branka Pejić^1^, Vladimir A. Trifonov^2^, Mladen Vujošević^1^, Jelena Blagojević^1^

#### ^1^Department of Genetics, Institute for Biological Research “Siniša Stanković”, University of Belgrade, Serbia; ^2^Department of the Diversity and Evolution of Genomes, Institute of Molecular and Cellular Biology Siberian Branch of the Russian Academy of Sciences, Novosibirsk, Russia

**Correspondence:** Marija Rajičić (marija.rajicic@ibiss.bg.ac.rs)

**Background**

Nearly two percent of karyotyped Mammalian species possess B chromosomes (Bs). Presence of Bs in even one third of species in the genus *Apodemus* makes this genus the headmost among mammalian Bs-carriers. Continuous studies revealed presence of Bs in yellow-necked mouse, *Apodemus flavicollis,* almost everywhere through its areal in different frequencies. It is established that frequency of animals with Bs in populations is correlated with certain biological and ecological variables. Their presence influence development of some morphometric characters which could be beneficial to B carriers under variable environmental conditions. Recent studies have revealed the potential origin and genetic composition of supernumeraries in this species. Sequences of Bs in *A. flavicollis* are enriched with cell cycle and microtubule protein-coding genes. Some of these genes are disrupted by multiple missense substitutions, which make them B-specific. Knowledge of Bs molecular structure, transcriptional activity, and phenotypic effects allows a deeper understanding of their biological importance.

**Materials and Methods**

We studied the transcriptional activity of sequences identified on Bs, using RT-PCR. Presence and amount of mRNA transcribed from three genes: *Rraga*, *Haus6* and *Cenpe*, were analyzed in young animals spleen tissues.

**Results**

In contrast to the long-lasting opinion that Bs are genetically inert elements, our results demonstrate that B-specific pseudogenes are transcribed in this species. Furthermore, intact Bs-genes, or genes on the standard genome in the presence of Bs, show the higher level of transcriptional activity in a sex-dependent manner. Apart from sex, this transcriptional activity is linked with the age of the animals and the number of Bs.

**Conclusions**

Bs of *A. flavicollis* contribute to the transcriptome of their carriers by transcription of B specific genes and/or by regulation of genes from the standard genome. This indicates a considerable biological effect of supernumeraries for their carriers.

**Acknowledgments**

This research was funded by the Ministry of Education, Science and Technological Development of the Republic of Serbia, grant OI173003, Mladen Vujošević.

## O3-02 The impact of omics analyses in the modern view of B chromosomes

### Syed F Ahmad^1^, Diogo C Cabral-de-Mello^2^, Patrícia P Parise-Maltempi^2^, Vladimir P Margarido^3^, Guilherme T Valente^4^, Cesar Martins^1^

#### ^1^Department of Morphology, Institute of Biosciences at Botucatu, São Paulo State University (UNESP), Botucatu, SP, Brazil; ^2^Department of Biology, Institute of Biosciences at Rio Claro, São Paulo State University (UNESP), Rio Claro, SP, Brazil; ^3^Center of Biology Sciences and Health, West Paraná State University (UNIOESTE), Cascavel, PR, Brazil; ^4^Department of Bioprocess and Biotechnology, Agronomic Science School, São Paulo State University (UNESP), Botucatu, SP, Brazil.

**Correspondence:** Syed F Ahmad (farhan.phd.unesp@gmail.com)

**Background**

B chromosomes (Bs), a type of supernumerary chromosomes, are extra karyotypic units in addition to A (autosome) chromosomes and found in all major eukaryotic taxa. Bs are uniquely characterized due to their non-Mendelian inheritance, and represent an example of the genomic conflict. Over the decades, their genetic composition, function and evolution have remained an unresolved query, although a few successful attempts have been made to address these phenomena. A classical concept is that Bs are selfish and abundant with DNA repeats and transposons and, in most cases, they do not carry any function. However, recently, many proteins coding and transcribing genes were found on Bs in a variety of species throughout next generation sequencing.

**Materials and Methods**

Here we report a comprehensive list of previously reported B chromosome genes described in fungi, plants, insects, fish and mammals and add to this list a number of genes present on B chromosomes which we recently discovered in our fours models species, the tetra fishes *Astyanax mexicanus* and *Astyanax correntinus*, the cichlid fish *Astatotilapia latifasciata*, and the grasshopper *Abracris flavolineata.* We have generated high coverage next generation sequencing (NGS) datasets using Hi-seq Illumina technology for all the aforementioned organisms including samples with B chromosome (B+) and without B chromosome (B-).

**Results**

The comparative NGS analyses detected thousands of genes fragments as well as a few complete genes to be present on the Bs. The functional annotation revealed that the Bs have accumulated copies of many genes coding for diverse set of functions related to important biological phenomena such as cellular processes, metabolism, development, response to stimulus, immune response, localization, morphogenesis and biological regulation. Our results showed that the Bs are also enriched with interesting set of genes associated to cell cycle and chromosome formation, which might be important for the survival and establishment of Bs in the cell. We also found that the Bs harbor different types of transposable elements (TEs) such as Tc1-pogo, hobo-activator, and Gypsy, L2/rex, Jockey retroelements. Finally, we identified origin and patterns of genomic evolution related to B chromosome.

**Conclusions**

Based on these findings, we hypothesize that the accumulation of genes on B might have played a key part in driving its transmission, escape, survival and maintenance inside the cell. The ongoing discoveries of new genes on B chromosomes open an exciting debate about their possible role in important biological tasks.

**Acknowledgements**

This research was funded by São Paulo Research Foundation – FAPESP, grant numbers 2014/16477-3 and 2015/16661-1, and National Counsel of Technological and Scientific Development - CNPq, grant number 305321/2015-3.

## O3-03 lncRNAs characterization in *A. latifasciata* and B effects over cell regulation molecules

### Érica Ramos^1^, Ivan R Wolf^2^, Guilherme T Valente^2^, Cesar Martins^1^

#### ^1^Department of Morphology, Institute of Biosciences at Botucatu, São Paulo State University (UNESP), Botucatu, SP, Brazil; ^2^Department of Bioprocess and Biotechnology, Agronomic Science School, São Paulo State University (UNESP), Botucatu, SP, Brazil

**Correspondence:** Érica Ramos (erica.ramos00@gmail.com)

**Background**

*Astatotilapia latifasciata* is an African cichlid fish that carries 0 to 2 B chromosomes in ~ 30% of the animals in the population that might be important elements to species evolution. Content of Bs include genes, repetitive elements and ncRNAs. BncRNA is a putative lncRNA enriched in Bs and differentially expressed in *A. latifasciata* B+ animals. As lncRNAs play major role in cell regulation, including chromosome activation/inactivation, B chromosome could be acting over it. Therefore, our objective was to elucidate the association of B chromosomes presence and lncRNAs differential expression to better understand B effects over cell regulation.

**Materials and Methods**

We have analyzed RNAseq data of *A. latifasciata* animals with (B+) and without (B-) Bs. From those, we developed 2 independent approaches based in transcriptome (A1) and *de novo* assembly (A2). The A1 approach consisted in blasting *A. latifasciata* transcriptome to known lncRNAs sequences available in public databases. Next, we filtered the output list by differences in RNA expression levels when comparing B+ and B- animals. The selected sequences were submitted to RT-qPCR and differential expression was evaluated by t-test analysis. In the other hand, in A2 approach we performed *de novo* assembly to the raw RNAseq data (after filtering steps). The assembled sequences were filtered again by identity to bacterial, mitochondrial, other ncRNAs, transposable elements and known coding sequences. Also, we have eliminated redundant transcripts and sequences not present in *A. latifasciata* genome.

**Results**

*In silico analysis* of A1 resulted in 33 possible lncRNAs and only one, 7SL lncRNA, was upregulated in gonads of B+ males. Contrary to *in silico* analysis, *in vitro* tests revealed that only part of this lncRNA is possibly upregulated in gonads of B+ females and presented high identity to two 7SLRNA derived small RNAs, described in human. Parallel to this, A2 approach results showed a set of 268,729 candidate lncRNA transcripts to future expression profile analysis.

**Conclusions**

The findings reinforce the importance of describing *A. latifasciata* set of lncRNAs, since most of lncRNAs were not found in public databases due to low conservation. Considering that, we intend to work in refining the dataset. Moreover, we described two small RNAs derived from 7SL RNA that are probably upregulated in B+ female gonads, which is a clue to understand the ways Bs could act in female reproductive cells.

**Acknowledgements**

This research was funded by FAPESP (2016/10141-9 and 2015/16661-1) and CAPES.

## O3-04 B chromosome contains a PIWI-Interacting RNA (piRNA) machinery that maintains the genomic integrity

### Jordana I N Oliveira, Cesar Martins

#### Department of Morphology, Institute of Biosciences at Botucatu, São Paulo State University (UNESP), Botucatu, SP, Brazil

**Correspondence:** Jordana I N Oliveira (jordana.oliveira@unesp.br)

**Background**

B chromosomes (Bs) are supernumerary elements and their constitution, morphology and number vary among organisms. The B in *Astatotilapia latifasciata* (Cichlidae) is present in both sexes and is composed to pseudogenes, long-non coding RNAs, coding genes and transposable elements (TEs). In gonads, TEs are controlled by piRNA, a small non-coding RNA transcribed from clusters of sequences originated from TEs “junkyards”. PiRNAs ensure the genome integrity against new TE insertions. The aim of this study was to investigate the piRNAs role under the B chromosome presence, since this chromosome is enriched with TEs.

**Materials and Methods**

The *A. latifasciata* piRNome was constructed using proTRAC.pl pipeline based on small RNAseq, RepeatMasker, transcriptome and genome annotation data. Looking for piRNAs, B+ genomic regions (B blocks – B+ genomic regions with higher coverage of sequencing reads comparing to the B- genome) were subject to prediction of piRNAs clusters. Further, a B coding gene list was investigated to identify piRNA related genes. The B genes were validated by qPCR using primers with B specific mutations. The piRNA clusters and gene expression were validated by qRT-PCR using RNA from B- or B+ gonads.

**Results**

Three clusters located into B blocks were identified, these clusters were called *biwi1*, *biwi2*, *biwi3* and are enriched by degenerated TEs. *Biwi1* and *biwi3* are expressed only in B+ samples (males and females), and *biwi2* is upregulated in B+ samples. *Biwi1* has high similarity with BEL32-I retrotransposon. Previous studies already reported this element enriched in the B chromosome, but its transcription was not detected, suggesting that *biwi1* from the B could be controlling the BEL32-I element. Additionally, a piRNA biogenesis gene was found on the B gene list. This gene, “mitochondrial cardiolipin hidrolase” (*pld6*), has B specific mutations. The *pld6* expression is also upregulated in the B+ samples.

**Conclusions**

Our data suggest that the accumulation of B+ TEs seems to be co-evolving with piRNA control, also under the control of B *pld6* gene copy in the *A. latifasciata*. Thus, the B could be working to benefit the genome integrity against TEs mobilization, as a genome guardian.

**Acknowledgements**

This research was funded by São Paulo Research Foundation - FAPESP, grant numbers 2017/25193-7 and 2015/16661-1.

## Session 4 - Population genetics of B chromosomes

### S4-01 Are there B-chromosomes in human?

#### Ahmed Al-Rikabi, Thomas Liehr

##### Jena University Hospital, Friedrich Schiller University, Institute of Human Genetics, Am Klinikum 1, D-07747 Jena, Germany

**Correspondence:** Ahmed Al-Rikabi (Ahmed.Al-Rikabi@med.uni-jena.de)

B-chromosomes (Bs) are not described for human karyotype; still, there is a similar phenomenon observable there: small supernumerary marker chromosomes (sSMC). Both Bs and sSMC represent a heterogeneous collection of chromosomes added to the typical karyotype, and which are both small in size. They may consist of heterochromatic and/or euchromatic material. Also a predominance of maternal transmission was reported for both groups. Even though sSMC and Bs show some similarity it is still an open question if Bs are present among the heterogeneous group of sSMC. According to current theories, sSMC would need drive, drift or beneficial effects to increase in frequency in order to become B chromosome. Here we discuss if among sSMCs Bs might be hidden. We present three potential candidates which may already be, or may in future evolve into B chromosomes in human: (i) sSMC cases where the marker is stainable only by DNA derived from itself; (ii) acrocentric-derived inverted duplication sSMC without associated clinical phenotype; (iii) sSMC derived from different chromosomes by chromothripsis. Overall, the majority of sSMC are not to be considered as B-chromosomes. Nonetheless, a minority of sSMC show similarities to B-chromosomes. Further studies are necessary to come to final conclusions for that problem.

### S4-02 On the way to the modern B chromosomes: origin, generation, further evolution, and maintenance in populations

#### Nikolay B Rubtsov^1,2^, Ilyas Ye Jetybayev^1,3^, Alexander G Bugrov^2,3^

##### ^1^The Federal Research Center Institute of Cytology and Genetics, Russian Academy of Sciences, Novosibirsk, Russia; ^2^Novosibirsk State University, Novosibirsk, Russia; ^3^Institute of Systematics and Ecology of Animals, Russian Academy of Sciences, Novosibirsk, Russia

**Correspondence:** Nikolay B Rubtsov (rubt@bionet.nsc.ru)

B chromosomes were described in species from almost all taxa of eukaryotes. We compared size, morphology, and DNA content of the B chromosomes (Bs) of mammalian and Orthopteran species. In many species, evolution of the Bs led to formation of numerous morphotypes. Only in *Eyprepocnemis plorans* more than 70 morphotypes of Bs were described. Many different morphotypes of the Bs were also revealed in Korean field mouse, *Apodemus peninsulae*. However, there are fundamental differences between these Bs. However, despite different mechanisms of origination, further evolution, and difference of transitional forms of the Bs in grasshoppers and mammals, their final form appeared to be similar. According to the widespread view the major source of Bs are small proximal regions of A chromosomes. The initial Bs accumulate repetitive sequences, through insertions of DNA fragments accompanied by DNA amplification. In the advanced stages of this process Bs are usually C-positive and vary in size. Morphotypes and DNA content of the Bs on *A. peninsulae* are in a good agreement with this suggestion. However, in grasshoppers there are Bs containing large C-negative regions. In *E. plorans* and *Podisma sapporensis* they were studied thoroughly. Insertion of large C-negative region into C-positive Bs is highly unlikely. Here we propose the mechanism of the B chromosome formation through autosome degradation. On the first stage, in euchromatic region of autosome arm numerous clusters of repeats and then C-positive blocks were developed. Transcriptional activity of the genes located between them were modified. The frequency of meiotic abnormalities was expected to be increased leading to additional copy of this chromosome in descendants. On the next stage, these extra chromosome containing intercalary C-positive blocks regions undergo through deletions of euchromatic regions located between them. Small C-positive regions merged forming larger C-positive regions. This process probably starts from the proximal part of the chromosome and proceed eventually to highly C-positive B chromosomes with interstitial C-negative regions or C-negative regions in distal part. Amplification and insertion of new repeats also can contribute to B chromosome evolution. Similar mechanism of chromosome degradation was recently described for neo-Y-chromosome evolution in two phylogenetic lines in Pamphagidae grasshoppers. Suggested hypothesis is supported with findings of numerous intercalary C-blocks in S10 autosome of *E. plorans* and diversity of B chromosome morphotypes in *E. plorans* and *P. sapporensis.*

## O4-01 Analyses of cytotype and genetic variations in association with geographical distributions of B chromosome aneuploids in *Lilium amabile* Palibin

### Jong-Hwa Kim^1^, Yoon-Jung Hwang^2^, Eun-Chae Kwon^3^, Nam-Soo Kim^3,4^

#### ^1^Department of Horticulture, Kangwon National University, Chuncheon, 24341, Korea; ^2^Department of Biology, Shamyook University, Seoul, 01795, Korea; ^3^Department of Molecular Bioscience, Kangwon National University, 24341, Korea; ^4^Institute of Biomedical Sciences, Kangwon National University, Chuncheon, 24341, Korea

**Correspondence:** Nam-Soo Kim (kimnamsu@kangwon.ac.kr)

**Background**

Lilies are perennial herbaceous flowering plants and the genus *Lilium* contains more than 110 species. B chromosomes are known as many as in 33 *Lilium* species. *L. amabile* (2*n*=2*x*=24), an endemic lily in Korean peninsula, is such a species having B chromosomes. The extraordinarily large genomes of *Lilium* species were known be mostly comprised of LTR-retrotransposons and other types of repeats. Both B chromosomes and transposable elements share similar property as selfish nature to the host species. Thus, the current research was conducted to see the relationship between cytotype variations by B chromosome constitutions and genetic diversity measured by IRAP (Inter-Retrotransposon Amplified Polymorphisms) as well as the geographic distribution of the B chromosome accessions of *L. amabile*.

**Materials and Methods**

We collected 135 *L. amabile* accessions from 6 geographical sites throughout the Korean peninsula. Chromosome counting and karyotype were done with root-tips cells by conventional aceto-carmine staining (1%) and FISH using ribosomal RNA probes. IRAP analysis was performed according to the protocol described by Lee et al. (2016; Genes Genom 38: 467-477; Doi:10.1007/s13258-016-0398-2). Phylogenetic analysis was carried out using NTSYSpc version 3.2 (http://www.exetersoftware.com/cat/ntsyspc/ntsyspc.html). Population structure was analyzed with STRUCTURE version 2.3 (https://web.stanford.edu/group/pritchardlab/structure.html). The POPGERNE version 3.2 (http:/www.ualberta.ca/~fyeh/fyeh/) was used to calculate the Nei’s genetic diversity (H), polymorphic index, Shannon’s information index (SI), and effective number of alleles. The Arlequin 3.5 program (http://cmpg.unibe.ch/software/arlequin35/) was used to calculate the F_st_ and Tajima’s D values.

**Results**

Based on the size, there were two kinds of B chromosomes; large B and small b. The *L. amabile* accessions were classified into 11 different cytotypes as 24, 24+1B, 24+1B+1b, 24+1B+2b, 24+2B, 24+2B+1b, 24+2B+2b, 24+2B+4b, 24+2B+5b, 24+1b, and 24+2b. Diploids and different types of extra B or b chromosome aneuploids co-existed at all collection sites, such that the occurrence of the extra B or b chromosome was random and distributed throughout the collection sites in Korea. IRAP fingerprinting revealed a high degree of polymorphism among the accessions of *L. amabile.* Diversity indices of effective number of alleles, Nei’s diversity, and Shannon index did not differ significantly between *L. amabile* populations of diploids and aneuploids. The Tajima’s D neutrality value was slightly higher in the aneuploids compared to the diploids. Allele frequency differences between *L. amabile* populations of diploids and aneuploids were very low, as indicated by an F_ST_ value of 0.0027. *L. amabile* accessions were structured into 9 groups according to structure analysis and each group in the structure consisted of both diploids and aneuploids, implying that the grouping in the structure analysis was not directly related to the chromosome constitutions. *L. amabile* accessions were also classified into 9 clusters in phylogenetic analysis, and the clustering patterns generally corresponded to the grouping pattern in the structure analysis at the dip node in the dendrogram.

**Conclusions**

The B chromosome cytotypes were highly variable and the occurrences of different cytotypes were random among the 6 geographic populations. IRAP diversity was not related to the cytological diversities of diploid and aneuploids among *L. amabile* accessions, and genetic differentiation was not obvious. The geographical distribution of *L. amabile* was not related to either IRAP diversity or cytological diversity, implying that IRAP diversification predated *L. amabile* dispersion in Korea without genetic differentiation.

**Acknowledgements**

This work was carried out with a grant from National Research Foundation of Korea (https://www.nrf.re.kr/index; Project No.C1013144-01-01) to NSK and GSP project (213007-05-3-SBM10) from Korea Institute of Planning and Evaluation for Technology (IPET) to JHK.

## O4-02 Cytogenomics highlights the molecular evolution of the B in the grasshopper *Abracris flavolineata* at population level

### Ana Elisa Gasparotto, Diogo Milani, Diogo C Cabral-de-Mello

#### Department of Biology, Institute of Biosciences at Rio Claro, São Paulo State University (UNESP), Rio Claro, SP, Brazil

**Correspondence:** Ana Elisa Gasparotto (aelisag.16@gmail.com)

**Background**

B chromosomes are additional parasitic elements that have incapacity of pairing with standard A chromosomes and maintain themselves trough generations possible due to drive mechanisms. These elements are present in some individuals from some populations in distinct species presenting different number and variability. The most accepted hypothesis of its origin is that they have probably arisen from the A chromosomes but follow their own evolutionary pathway.

**Materials and Methods**

Here, we analyzed the presence, frequency, molecular composition and evolution of B chromosomes in five populations of the grasshopper *Abracris flavolineata*. For that, we estimated abundance of repetitive elements analyzing next generation sequencing (NGS) datasets from Illumina Hiseq technology obtained from individuals belonging to distinct populations harboring B chromosomes. In addition some selected repetitive DNAs were mapped through Fluorescent *in situ* Hybridization (FISH).

**Results**

Four of five populations reveal the occurrence of one B chromosome, except St. Barbara/PA. None of the populations presented individuals with two B chromosomes, as reported in Rio Claro/SP, suggesting differential drive and accumulation. Differently of all populations with a C-negative pattern on B chromosome, for Posadas/ARG the C-banding showed C-positive pattern along the entire B chromosome, indicating its heterochromatic nature. Genome data showed almost the same level of abundance and divergence for all repeats analyzed in +B genomes from Rio Claro/SP and Cabo/PE, with exception of U2 snDNA. Moreover for Posadas/ARG, only satDNAs presented higher abundance and low divergence. FISH mapping showed sharing signal of the U2 snDNA gene between the B chromosome and the largest autosomal pair for all populations, with the exception of Posadas/ARG, pointing a possible different B origin and composition. For other repeats such as satellites Afsat1, Afsat52 (satDNA) and transposable elements Af_Jockey5 and Af_Gypsy12 (TE), the B chromosome of Posadas/ARG showed intense accumulation of satDNA and lack/small signals of TEs. However, for all other populations, the TEs showed the similar pattern of Rio Claro/SP, which is spread in euchromatic regions on the chromosomal arms and accumulation of satDNA in centromeric and telomeric regions.

**Conclusions**

Our findings elucidate questions about the different rates of accumulation, evolution and composition of the B chromosome between populations. The data highlight different patterns of accumulation and composition between Bs at population level. Moreover we suggest a new type of variant regarding its distinctive composition in Posadas/ARG.

**Acknowledgements**

FAPESP-2015/16661-1, CAPES and CNPq funded this research.

## P4-01 Interpopulational variation in the rodent *Akodon montensis* due to the presence of B chromosomes

### Letícia R Cruz, Valéria Fagundes

#### Laboratory of Animal Genetics (LGA), Department of Biological Science, Federal University of the Espírito Santo State (UFES), ES, Brazil

**Correspondence:** Letícia R Cruz (leticiarosariocruz@gmail.com)

**Background**

*Akodon montensis* is a neotropical rodent species that occurs in the Atlantic Rainforest from Eastern Paraguay and Northern Argentina throughout southern Brazil until northern Minas Gerais. This species has 2n=24 as a basic diploid number. Some variations due to monosomy of the X chromosome (2n=23) and presence of 1-3 B chromosomes (2n=25-27) were reported. Previous analyses led us to suggest that the presence of B is a population-specific variation, but a comprehensive revision as well as increasing of sample is need to better understand these variations.

**Materials and Methods**

We analyzed the karyotype (bone marrow preparations) of 107 individuals from Minas Gerais, São Paulo, Paraná and Rio Grande do Sul in Brazil. To carry out a literature review, we look for published papers in indexed platforms (Google Scholar and Scielo) and unpublished documents as theses, dissertations and undergraduate theses that showed at least one picture and karyotype description.

**Results**

Our data revealed presence of one B (2n=25) in 14,3% from Minas Gerais sample (n=7) and in 14,5% of São Paulo's (n=83). Only one specimen from São Paulo (1,2%) had two Bs (2n=26). No B was found on Paraná (n=3) and Rio Grande do Sul (n=14). The revision survey recovered 446 records, that summed to our sample, resumed 553 specimens. From Minas Gerais (n=9) only one specimen had B from Caxambu. From São Paulo (n=315), 17,1% presented 2n=25 and one specimen (0,3%) from Buri showed 2n=26. From Paraná (n=111), 4,5% showed 2n=25 and 1,8% showed 2n=26. From Santa Catarina (n=22), 54,4% showed 2n=25, 13,6% showed 2n=26 and one specimen (4,5%) showed 2n=27. In Argentina (n=35), 17,1% showed 2n=25. From Rio Grande do Sul (n=47) and Rio de Janeiro (n=14) no specimens presented B.

**Conclusions**

Overall, the presence of chromosome B among populations/regions was widely distributed, except for two states, including high altitudes and at sea level regions. However, since each region presented a specific and variable frequency of B, our analysis corroborated the previous assumption that the presence of supernumerary chromosome in *A. montensis* has an interpopulational variation.

**Acknowledgements**

This research was funded by Espírito Santo Research Foundation - FAPES (grant number 800600417/17 to VF) and National Counsel of Technological and Scientific Development - CNPq (undergraduate scholarship to LRC).

## Session 5 - Segregation behavior and drive of B chromosomes.

### S5-01 B chromosomes - a matter of chromosome drive

#### DanDan Wu, Alevtina Ruban, Jörg Fuchs, Anastassia Boudichevskaia, Ali M Banaei Moghaddam, Veit Schubert, Daiyan Li, Andreas Houben

##### Leibniz Institute of Plant Genetics and Crop Plant Research (IPK), Gatersleben, 06466 Stadt Seeland, Germany

**Correspondence:** Andreas Houben (houben@ipk-gatersleben.de)

**Background**

Often the transmission rate of B chromosomes (Bs) is higher than 0.5, not obeying the Mendelian law of equal segregation. The resulting transmission advantage is collectively referred to as ‘drive’. The maximum number of Bs tolerated by the host varies between species and depends on a balance between the B chromosome drive, and the negative effects, especially on fertility and vigour, caused by B chromosomes if they occur in higher numbers. There is a variety of mechanisms of B chromosome drive that occurs before, during or after meiosis, while in some cases drive has not been found.

**Results and Conclusions**

Here we analyzed the B chromosome drive of the grasses *Aegilops speltoides* and *Secale cereale* and provide direct insight into its cellular mechanism. We first employed comparative genomics to identify a B-specific repeat, which we used to track the B chromosomes during microgametogenesis. It was found that sister chromatid nondisjunction and an asymmetric spindle during first pollen grain mitosis are components of the B chromosome accumulation process. A different centromere activity of B chromosomes could be excluded as the reason for the nondisjunction of Bs, since the centromeres of all chromosomes are CENH3-positive and interact with tubulin. To determine the B accumulation rate in sperm nuclei, we developed a novel flow cytometric approach that allowed the differentiation between vegetative and sperm nuclei as well as the quantification of B chromosomes inside the nuclei. Utilizing this method, we found that independent of the number of B chromosomes present in the mother plant, Bs accumulate in the generative nuclei to more than 93% in *Ae. speltoides*. Based on a comparative RNA-seq analysis we could identify rye B-specific transcripts associated with the process of chromosome drive. The prerequisites for the drive process seems to be common in Poaceae, thus enabling independent origins of Bs in different lineages within the family.

**Acknowledgements**

This research was funded by the Deutsche Forschungsgemeinschaft (HO 1779/30-1) and the China Scholarship Council.

### S5-02 Studies on the mitotic drive mechanism of the maize B chromosome facilitated by sequence data

#### Changzeng Zhao, Hua Yang, Patrice Albert, James A Birchler

##### Division of Biological Sciences, University of Missouri, Columbia, MO 65211 USA

**Correspondence:** James Birchler (BirchlerJ@missouri.edu)

**Background**

The B chromosome of maize is a nonvital chromosome but is maintained in populations by an accumulation mechanism. This mechanism consists of nondisjunction at the second pollen mitosis coupled with preferential fertilization of the egg by the sperm with the B chromosomes, as opposed to the polar nuclei, in the process of double fertilization. The nondisjunction property is known to reside at or near the centromere and requires other regions of the chromosome that act in trans, most notably the terminus of the long arm. The centromere of the B chromosome contains the canonical centromeric repeats of maize but also a B chromosome specific repeat that is interspersed within and around the centromere spanning several megabases. The sequence of the maize B chromosome is nearing completion by an international group (See other contributions) and this information was used to guide an understanding of the accumulation mechanism.

**Materials and Methods**

Translocations between the B chromosome and various A chromosome arms were used to locate the breakpoints on the B chromosome, of particular note in the centromeric region. Copies of the B centromere specific repeat unit were isolated and ligated together to form multimers. The product of these reactions were co-bombarded with a selectable marker into maize embryos for transformation. Selected transformations were tested as to whether they could induce an attempted nondisjunction at the insert site when combined with normal B chromosomes to supply the trans-acting factor(s). This would cause chromosomal breaks that could be documented by crossing them to phenotypic markers that would allow recognition of such events. The localization of B-A translocation breakpoints on the B chromosome also allowed a narrowing of the region of the B chromosome that is necessary for preferential fertilization.

**Results**

Conglomerates of the B specific centromere repeat were recovered in several chromosomes arms but were analyzed for 4L, 5S, and 7S most thoroughly. Control crosses to the respective testers with the transformed conglomerates do not provide evidence of chromosomal breakage alone nor do crosses with a line with normal B chromosomes. However, when the transformed inserts are combined with full sized B chromosomes, mosaic kernels were found well above the background level. With regard to the other aspect of the accumulation mechanism, analysis of the most proximally broken B-A translocations indicates that the B-A chromosome fosters preferential fertilization as evidenced by the skewing of non-concordant kernels in favor of embryo fertilizations. This results suggests that the centromeric region is the component of the B chromosome involved with preferential fertilization.

**Conclusions**

Despite the fact that the conglomerate repeat insertions are much smaller than the B specific repeat array in the B chromosome, they foster chromosomal breaks at the second pollen mitosis for the chromosome arm in which they reside. This observation provides evidence that the B specific repeat conditions the nondisjunction property of the B chromosome. Because the centromeric region is also implicated in preferential fertilization, the B specific centromeric repeat is a likely candidate as the responsible sequence for this property of the B chromosome as well and potentially unifies the two aspects of the accumulation mechanism.

**Acknowledgements**

This research was funded by National Science Foundation grants IOS-1444514 and IOS-1545780.

### S5-03 Meiotic drive of B chromosomes in *Drosophila melanogaster*

#### Stacey L Hanlon^1^, R Scott Hawley^1,2^

##### ^1^Stowers Institute for Medical Research, Kansas City, MO, USA; ^2^ Department of Molecular and Integrative Physiology, University of Kansas Medical Center, Kansas City, KS, USA

**Correspondence:** Stacey L Hanlon (slh@stowers.org)

**Background**

Hundreds of species have been found to carry B chromosomes, including nine that are within the genus *Drosophila*. The most recent addition to this group was *D. melanogaster*, where a single laboratory stock was found to carry 10-12 B chromosomes. Our molecular characterization of the *D. melanogaster* B chromosome revealed that it has a centromere, telomeres, and no known genic regions, leaving us to suspect its accumulation within this stock may be due to a drive mechanism.

**Materials and Methods**

The B chromosomes were found in a single stock that carries both a mutation in *matrimony*, which is a Polo kinase inhibitor specific to female meiosis, and a rearranged balancer chromosome that enables the maintenance of the *matrimony* mutation in the stock. To investigate the inheritance pattern of the B chromosomes, we measured their transmission frequency from individual parents to progeny. Single, unmated individuals carrying B chromosomes were crossed to a wild-type stock that does not carry B chromosomes. The number of B chromosomes in both the parents and progeny were determined cytologically from metaphase chromosome spreads made from adult gonad tissue squashes. This method enabled us to calculate the transmission frequency of B chromosomes from a single parent to a single progeny.

**Results**

We found that in their endogenous stock background, the *D. melanogaster* B chromosomes show an elevated transmission frequency when passed through the female parent but not through the male parent, indicating the B chromosomes are subject to meiotic drive that is female-specific. Both the *matrimony* mutation and the balancer chromosome each contribute to this female meiotic drive and, based on our examination of oocytes arrested in meiotic metaphase I, we hypothesize each can disrupt the spatial arrangement of the B chromosomes within the oocyte.

**Conclusions**

Our work with the B chromosomes in *D. melanogaster* has revealed a novel female meiotic drive system, where the biased inheritance of the B chromosomes is a product of the genetic context of the individual rather than through a mechanism supplied by the selfish element itself. Due to the role of *matrimony* in the drive of the B chromosomes and its rapid evolution within the *Drosophila* genus, our work provides fresh insight into why meiotic genes are some of the most rapidly evolving in the genome.

**Acknowledgements**

This work was made possible by the generous support of the Stowers Institute through an endowment by Jim and Virginia Stowers.

### S5-04 Meiotic drive of female-inherited accessory chromosomes in a plant pathogenic fungus

#### Michael Habig^1^, Hannah Justen^1^, Mareike Möller^1^, Jakob Quade^1^, Gert Kema^2^, Eva H Stukenbrock^1^

##### ^1^Christian-Albrechts University of Kiel, Environmental Genomics, Kiel, Germany; Max Planck Institute for Evolutionary Biology, Plön, Germany; ^2^Plant Research International BV, Wageningen, the Netherlands

**Correspondence:** Michael Habig (mhabig@bot.uni-kiel.de)

**Background**

Accessory chromosomes are present in a variety of fungal pathogens where they can directly affect host-specificity and pathogenicity. The genome of the important fungal wheat pathogen *Zymoseptoria tritici* comprises an exceptionally large complement of up to eight distinct accessory chromosomes ranging in size from 0.4 to 0.8 Mb. How and why this large complement of accessory chromosomes is maintained is unknown. Here, we address the functional relevance and mode of transmission of accessory chromosomes during mitotic and meiotic cell divisions in *Z. tritici*. We show that the presence of accessory chromosomes is associated with a fitness cost *in planta*. Furthermore, accessory chromosomes, which are enriched with the histone modification H3K27me3, are frequently lost during mitotic cell divisions. On the other hand, using mating experiments, we demonstrate that accessory chromosomes increase in frequency during meiosis. *Z. tritici* has a heterothallic mating system, ie. two haploid individuals of different mating type are required to form a diploid zygote, which undergoes meiosis. If the two parental individuals differ in their complement of accessory chromosomes, the resulting diploid zygote will contain unpaired chromosomes lacking a homolog. Here, we show that unpaired chromosomes inherited from the female parental individual are transmitted to all the meiotic products instead of the expected half. Paired accessory chromosomes and male-inherited unpaired accessory chromosomes, however, largely follow Mendelian inheritance. This represents a novel meiotic drive mechanism for fungal accessory chromosomes. We speculate that the accessory chromosomes of *Z. tritici* represent a novel group of selfish genetic elements.

## O5-01 Catching transcripts involved in the process of B chromosome drive

### Anastassia Boudichevskaia^1^, Andrea Bräutigam^2^, Andreas Houben^1^

#### ^1^Leibniz Institute of Plant Genetics and Crop Plant Research (IPK), Gatersleben, Germany; ^2^Fakultät für Biologie, Bielefeld University, 33615 Bielefeld, Germany

**Correspondence:** Anastassia Boudichevskaia (boudichevskaia@ipk-gatersleben.de)

**Background**

Drive is the main feature of B chromosomes (Bs) in certain species of plants. In Gramineae, the chromosome drive involves directed nondisjunction of Bs at first pollen mitosis, which allows the Bs to be transmitted at greater rate than Mendelian frequency. Thus far, however no gene, or other nondisjunction element, has been characterized that plays a role in the B accumulation mechanism. In order to identify transcripts controlling the process of drive we used the B chromosome of rye (*Secale cereale*) as a model. The B of rye we analyzed exists in the background of wheat to distinguish between transcriptomes of the B and its host. We intent to measure and compare the transcripts of pollen undergoing the first pollen mitosis with and without different variants of Bs. We aim to identify trans-acting coding and/or noncoding transcripts, involved in the regulation of B chromosome drive and to characterize the identified candidate sequences. This analysis will provide new evidence regarding the generation of rapid genome changes in higher eukaryotes, with particular relevance to selfish chromosomes, and will improve our knowledge of the mechanism underlying the segregation behaviour of chromosomes.

**Materials and Methods**

We report a rye B-specific transcriptome, produced by *de novo* assembly of RNA sequencing reads. Data were obtained from plants of hexaploid wheat cv Chinese Spring without rye Bs (0B), with added rye standard Bs and a variant of rye Bs, called B deficient chromosome (Bdef). The Bdef variant is characterized by the loss of the nondisjunction control region and absence of drive. We characterized and annotated the B specific transcriptome. A number of analytical steps based on the comparison of the standard Bs with Bdef transcriptome profile allowed us to identify transcript isoforms localized in the nondisjunction control region. In addition, we analyzed how the number of Bs affects the gene expression pattern and whether the rye B can influence the expression of wheat-linked genes.

**Results**

We categorized the candidate B-specific transcripts in four classes based on their functional annotation: transposons (e.g. reverse transcriptase, transposase); potential transposons (e.g. zinc finger, aspartic protease) and unknown and canonical genes with specified functions. GO term enrichment analysis of the B specific transcript isoforms revealed that terms chromosome (GO:0005694), chromosomal part (GO:0044427), cortical microtubule organization (GO:0043622), kinetochore (GO:0000776), and microtubule binding (GO:0008017) are significantly overrepresented. To verify the specificity of the identified nondisjunction control region candidate sequences we performed mapping against the microdissected DNA coming from the B nondisjunction control region.

**Conclusions**

Based on the comparative RNA-seq analysis we could identify rye B-specific transcripts involved in general chromatin- and chromosome-related processes. Bs transcript isoforms representing the nondisjunction control region could be considered as potential candidates involved in the regulation of B drive. Next, candidate genes will be tested to better understand the mechanism of B drive.

**Acknowledgements**

This research was funded by Deutsche Forschungsgemeinschaft (HO 1779/30-1). We are grateful to members of the IPK, Gatersleben, Germany: Katrin Kumke for excellent technical assistance; Anne Fiebig for supporting bioinformatics analysis; Axel Himmelbach for construction of cDNA libraries and sequencing; Thomas Schmutzer (Martin Luther University Halle-Wittenberg, Halle, Germany) for access to the rye B microdissected DNA.

## O5-02 *Aegilops speltoides* B chromosomes and the process of their organ-specific elimination.

### Alevtina Ruban^1^, Thomas Schmutzer^1,2^, Dan D. Wu^1,3^, Jiri Macas^4^, Jörg Fuchs^1^, Veit Schubert^1^, Uwe Scholz^1^, Andreas Houben^1^

#### ^1^Leibniz Institute of Plant Genetics and Crop Plant Research (IPK) Gatersleben, 06466 Seeland, Germany; ^2^Martin-Luther-University Halle-Wittenberg, Institute of Agricultural and Nutritional Sciences, 06099 Halle (Saale), Germany; ^3^Triticeae Research Institute, Sichuan Agricultural University, 611130 Wenjiang, China; ^4^Biology Centre, Czech Academy of Sciences, Ceske Budejovice, Czech Republic

**Correspondence:** Alevtina Ruban (ruban@ipk-gatersleben.de)

**Background**

B chromosomes (Bs), as an optional addition to the basic chromosome complement, do not affect significantly the host fitness and fertility when appear in low number. If present, Bs are usually constant within individual organisms. However, in some species, Bs demonstrate an organ-type specific distribution. Whether this is a result of genome instabilities caused by the presence of additional chromosomes, or a result of a specifically controlled process of chromosome elimination, is not known yet. Programmed chromosome elimination during the development of an organism has been reported for different animal species, but was never shown in plants. Here we investigated the process behind the root-specific absence of B chromosomes in the goat grass *Aegilops speltoides*. In this species Bs are present in all aerial organs but absent in roots. In addition, we determined the sequence composition and origin of *Ae. speltoides* Bs.

**Materials and Methods**

To identify B-specific repeats suitable as in situ hybridization markers we performed similarity-based clustering analysis of Illumina sequence reads. The analysis was run on the RepeatExplorer server (https://repeatexplorer-elixir.cerit-sc.cz/) in a comparative mode using the reads generated from genomic DNA of plants without Bs and with 2 Bs. B-specific or B-enriched repeats were then identified as clusters which were predominantly made of reads from 2B plants.

We adapted a tissue section protocol suitable for FISH and immunostaining analysis of developing seeds to visualize the process of B chromosome elimination. Tissue culture was employed to assess the B-specific elimination during induced rhizogenesis.

**Results**

We identified two highly abundant B-specific repeats and tracked the origin of single- and low copy B-located sequences back to the corresponding A chromosomes and organelles of *Ae. speltodies*. Using the newly found B-specific repeats, we were able to trace the B chromosomes during their elimination and determined how and at which stage of plant development the Bs become removed from the roots. We also have shown that B chromosomes undergo elimination independently of the root type.

**Conclusions**

Our results indicate that the root-specific elimination of Bs in *Ae. speltoides* is not random but a strictly controlled process which occurs at the beginning of root differentiation irrespective of the root type. Thus, programmed chromosome elimination exists also in plants. Why the Bs in *Ae. speltoides* are subjected to elimination in root tissue but retained in all other tissues is still a mystery. Considering the similarities between root-specific elimination of Bs in *Ae. speltoides* and some examples of programmed chromosome elimination from somatic tissue in animals, we suggest that the mechanism behind might be evolutionary conserved among a wide range of taxa, including plant species.

Beside the high copy sequences, we were also able to identify B-located gene-like sequences. The similarity of these sequences to those located on A chromosomes indicates that the B chromosome in this species is a mosaic product of host nuclear and organelle sequences which underwent rearrangements and amplification during the formation and evolution of Bs. Whether the gene-like sequences located on Bs are currently functional is still unknown.

**Acknowledgements**

This research was funded by Deutsche Forschungsgemeinschaft (HO1779/26-1, HO1779/30-1). Dan D. Wu was supported by China Scholarship Council (Grant No. 201606910015).

## O5-03 Flow cytometric estimation of the B chromosome transmission rate in pollen grains of the wild wheat *Aegilops speltoides*

### Joerg Fuchs^1^, Dandan Wu^1,2^, Alevtina Ruban^1,3^, Andreas Houben^1^

#### ^1^Leibniz Institute of Plant Genetics and Crop Plant Research Gatersleben (IPK), Stadt Seeland, Germany; ^2^Triticeae Institute, Sichuan Agricultural University, Chengdu, China; ^3^KWS Saat SE, Einbeck, Germany

**Correspondence:** Joerg Fuchs (fuchs@ipk-gatersleben.de)

**Background**

B chromosomes (Bs) are supernumerary chromosomes, which often show a non-Mendelian inheritance. Mitotic or meiotic alterations often lead to a transmission of Bs in higher-than-expected frequencies resulting in their accumulation in the progenies. This chromosome drive is one of the most important features of Bs. However, little is known about the mechanism behind the drive. Here we analyzed the drive of Bs in pollen grains of the wild wheat *Aegilops speltoides*.

**Materials and Methods**

Flow cytometric analysis of isolated pollen nuclei of A. speltoides allowed the differentiation between vegetative and sperm nuclei as well as the quantification of Bs inside the nuclei. A combination of these analyses with immunostaining and FISH on sorted nuclei of the obtained fractions enabled us to quantify the drive of Bs.

**Results**

The newly developed flow cytomtery-based approach to quantify the accumulation frequency of Bs during microgametogenesis revealed a highly efficient accumulation of Bs in generative nuclei during the first pollen mitosis. Independent of the number of Bs carried by the host (+1B to +6B were analysed), B chromosomes accumulate with more than 93%. Corresponding cytogenetic analysis of the first pollen mitosis using anther tissue sections confirmed the obtained data.

**Conclusions**

The proposed approach is an excellent method to estimate the transmission rate of B chromosomes during pollen mitosis and additionally allows conclusions regarding the preceding meiosis. Based on differences in the DNA content the number of transmitted B chromosomes can precisely be determined. In contrast to cytological approaches, it is much faster and much higher numbers of nuclei can be evaluated; however, formed micronuclei escape their detection so far.

**Acknowledgements**

This work was supported by the China Scholarship Council (Grant No. 201606910015) (to DW) and the Deutsche Forschungsgemeinschaft DFG (Grant No. HO1779/26-1) (to AH).

## O5-04 B chromosomes are preferentially expulsed in comparison to A chromosomes during spermatogenesis in *Abracris flavolineata*

### Lucas Albuquerque, Diogo Milani, Diogo C. Cabral-de-Mello

#### Department of Biology, Institute of Biosciences at Rio Claro, São Paulo State University (UNESP), Rio Claro, SP, Brazil

**Correspondence:** Lucas Albuquerque (lucasabq@live.com)

**Background**

The long-term evolution of B chromosomes in populations is compared to an arms race between the host genome and the parasitic element. Mechanisms involved in both fronts have been the focus of intriguing questions about genetic drive to transmission advantage, genome tolerance and the host responses to deleterious effects of an overload. Expulsion of B chromosomes is rarely documented. One of those reports is the preferential loss of B chromosomes due to unsuccessful gamete formation.

**Materials and Methods**

Here we tested the possibility of B chromosome expulsion analyzing the presence of B chromosomes in normal and abnormal spermatids of the grasshopper *Abracris flavolineata* by means of Fluorescence *in situ* Hybridization (FISH). For that we used as probes the U2 snDNA cluster that is enriched on B chromosomes and to test the possible expulsion of A genetic content we used Afsat1 probe.

**Results**

Individuals bearing one or two B chromosomes exhibited higher frequency of macro- and micro-spermatids in comparison to 0B animals. FISH analysis revealed 1B-males showing similar proportion of normal sized spermatids B-carrying and B-lacking (48% of B-carrying), according with Mendelian previsions (B transmission rate, kB, equals 0.5). Nevertheless, B frequency in normal spermatids from 2B-males (0.77) was significantly lower than Mendelian rate expectations, indicating a relief of a B overload by the deficit of 2B and increase of 1B cells, most likely due to B loss in the 2B spermatocytes. The preferential expulsion of the B chromosomes was documented by the B presence in 81,6% of the micro-spermatids, which did not contain A chromosomes in nearly all the cases (100 of 103 cases). Our results indicate a loss of 23% of B chromosomes from the sperms. We did not detect abnormalities during meiosis (like anaphase bridges), indicating that the B expulsion occurs post-meiotically.

**Conclusions**

Our results suggest a defensive mechanism by the genome in response to excessive accumulation of the B chromosomes. This defense occurs by preferential elimination of B chromosomes in regard to A chromosomes during spermatogenesis.

**Acknowledgements**

We acknowledge for financial support to CAPES and FAPESP (grant number 2015/16661-1).

## O5-05 Behavior of a holocentric chromosome during spermatogenesis

### Emiliano Martí, Vanessa Bellini Bardella, Diogo C Cabral-de-Mello

#### Department of Biology, Institute of Biosciences at Rio Claro, São Paulo State University (UNESP), Rio Claro, SP, Brazil

**Correspondence:** Emiliano Martí (emilianomarti1@gmail.com)

**Background**

B chromosomes, that are supernumerary elements, were discovered in a species with holocentric chromosomes. Although, our current knowledge about biology of B chromosomes were obtained mainly from species with monocentric chromosomes. Concerning behavior during cell divisions the two types of chromosomes (monocentric and holocentric) has distinct patterns of segregation, which putatively could influence the inheritance of the B chromosomes. Here we analyzed the behavior of the holocentric B chromosome of the hemipteran *Aetalion reticulatum*.

**Materials and Methods**

Adult males were collected in São Paulo State University Campus, Rio Claro/São Paulo, Brazil and testes were fixed in Carnoy solution. From previous analysis we selected two satellite DNAs to use as probes in Fluorescent *in situ* Hybridization (FISH) for tracking the A and B chromosomes during spermatogenesis.

**Results**

In order to analyze the meiotic behavior of the holocentric supernumerary element as well as its mitotic stability chromosomal preparations were made from each follicle separately from three individuals. Analysis of metaphase I revealed variation of number of Bs (from 0 to 3) at intra- and inter-follicular level, indicating mitotic instability for B chromosomes. During anaphase chromosomal bridges were frequently noticed, but in all cases, they were composed exclusively by A chromosome DNA (revealed by FISH mapping). Although, abnormal spermatids (macro and microspermatids) or micronucleus were not evidenced.

**Conclusions**

B chromosome analyzed here showed variation consistent with mitotic instability. In addition, our results indicate that the host genome would not have developed specific B chromosome expulsion mechanisms in any stage of spermatogenesis. As well known, holocentric chromosomes have a different behavior concerning segregation, and these features may facilitate the permanence of supernumerary elements in genomes. Finally, given the holocentric nature, the anaphase bridges, like observed in *A. reticulatum*, that could result in chromosomal fragments are sources for origin of new supernumerary elements.

**Acknowledgements**

FAPESP (grant number 2015/16661-1) has funded this research.

## P5-01 Characterization of mitotic nondisjunction and identification of the B chromosome nondisjunction regulatory region in the goat grass *Aegilops speltoides*

### Daiyan Li, Alevtina Ruban, Andreas Houben

#### Leibniz-Institute of Plant Genetics and Crop Plant Research (IPK), Gatersleben, 06466 Stadt Seeland, Germany

**Correspondence:** Daiyan Li (lid@ipk-gatersleben.de)

**Background**

B chromosomes (Bs) are supernumerary chromosomes that are often preferentially inherited. *Aegilops speltoides* is an annual diploid Poaceae with a maximum number of eight additional Bs besides the inherent seven pairs of standard A chromosomes. Nondisjunction of Bs occurs during the postmeiotic drive and the root tissue-specific elimination of Bs. Thus, *Ae. speltoides* serves as an ideal model for understanding what determines the process of nondisjunction. Unknown is, whether a nondisjunction control region exists in this species as been reported for the B chromosome of rye.

**Results**

Does the chromatin composition differ between A and B chromosomes in anaphase cells undergoing B chromosome elimination?

To investigate this question, a set of histone variant-specific antibodies will be employed to immunolabel young embryos of +B *Ae. speltoides* undergoing B chromosome elimination. The distribution of immunosignals along separated A chromatids and non-separate B chromatids will be compared. Next, post-translational histone modifications distinguishing between separated and non-separated chromatids will be used to immunolabel +B anaphase cells at first pollen mitosis isolated from *Ae. speltoides* and rye. Particularly, the status of phosphorylation of histone H3 will be tested, because this cell cycle dependent histone modification correlates with the process of sister chromatid cohesion in plants.

Searching for the regulatory region controlling nondisjunction of B chromatids in *Ae. speltoides*

For the identification of the nondisjunction regulatory region, we will employ X-ray mutagenesis to induce chromosome mutations in the pollen of +B *Ae. speltoides* plants. Mutagenized pollen will be used to pollinate 0B receptor plants for seed generation. In order to test whether the B chromosome conserved repeat AesTR-183 is involved in the control of nondisjunction, we aim to identify *Ae. speltoides* plants carrying a mutated B without the AesTR-183-positive chromosome region.

**Acknowledgments**

This research was funded by a fellowship granted to Daiyan Li and by the DFG (HO1779/30-1).

## P5-02 Meiotic behavior and transmission of the supernumerary B chromosome of the cichlid *Astatotilapia latifasciata*

### Adauto L Cardoso^1^, Natália B Venturelli^1^, Irene MC Güerisoli^2^, Rogério A Oliveira^3^, Ricardo Benavente^2^, Cesar Martins^1^

#### ^1^Department of Morphology, Institute of Biosciences at Botucatu, São Paulo State University, Botucatu, SP, Brazil; ^2^Department of Cell and Developmental Biology, Biocenter, University of Würzburg, Würzburg, Germany; ^3^Department of Biostatistic, Institute of Biosciences at Botucatu, São Paulo State University, Botucatu, SP, Brazil

**Correspondence:** Adauto L Cardoso (adautolimacardoso@gmail.com)

**Background**

Supernumerary B chromosomes (Bs) are dispensable genetic elements found in the genome of eukaryotic organisms. During meiosis Bs usually are distinct of A chromosome (regular set of chromosomes) in behavior and rate of transmission. Individuals of *Astatotilapia latifascita* (cichlid fish) can harbor 0 to 2 Bs, which have been explored with integrated approaches of cytogenetics, molecular biology, genomics and bioinformatics. The exploration of B behavior and transmission in this species can help to elucidate its mechanism of maintenance.

**Material and Methods**

Here we analyzed the meiotic behavior of Bs by conventional staining (Giemsa) and immunostaining using the antibodies medakaSYCP1 Guinea Pig (synaptonemal complex 1), medakaSYCP3 Rabbit (synaptonemal complex 3) and mouseSMC3 Rabbit (coesin complex). The results were analyzed by super resolution technology by structured illumination (SIM). Moreover, we performed different types of crosses (male without B x female without B, male without B x female with B, male with B x female without B and male with B x female with B) and analyzed the frequency of B chromosomes in the offspring using a multivariate logistic regression model in order to determine the contribution of males and females in B transmission to the offspring.

**Results**

In meiotic cells of individuals with a B chromosome we identified the presence of 22 bivalents relative to the 44 A chromosomes and 1 univalent that is equivalent to B. With immunostaining of pachytene cells we detected the presence of proteins related of assembling of synaptonemal complex in all bivalents and in the structure relative to the B chromosome. In our analyses of crosses we found that both males and females contribute to the offspring possess B, but the female contribution (odds ratio – OD = 2.47) is almost twice as large as the male contribution (OD = 1.7).

**Discussion**

Our findings indicate that the B chromosome of *A. latifasciata* perform self-pairing during meiosis, since we could observe the presence of synaptonemal complex elements associated with this chromosome, manly the central element. This can be supported by a previous theory that this B is an isochromosome. In turn, the analyses of crosses indicate an occurrence of drive mechanism specific to females, which can explain the maintenance of the B.

**Conclusions**

Here we found evidences of self-pairing of the B chromosome and a probable mechanism of drive specific to females.

**Acknowledgements**

This research was funded by São Paulo Research Foundation - FAPESP, grant numbers 2013/25234-4, 2015/16661-1 and 2017/07484-4, and National Counsel of Technological and Scientific Development - CNPq, grant number 305321/2015-3.

## Session 6 - New technologies and applications of B chromosomes

### S6-01 Leveraging long read sequencing technologies to assemble the selfish B chromosome in *Nasonia vitripennis*

#### Igor Antoshechkin^1^, Elena D Benetta^2,3^, Patrick M Ferree^2^, Omar S Akbari^3^

##### ^1^Division of Biology and Biological Engineering, California Institute of Technology, Pasadena, California, 91125, USA; ^2^W. M. Keck Science Department of Claremont McKenna, Pitzer, and Scripps Colleges, Claremont, California, 91711, USA; ^3^Division of Biological Sciences, Section of Cell and Developmental Biology, University of California, San Diego, La Jolla, California, 92093, USA

**Correspondence:** Igor Antoshechkin (igor.antoshechkin@caltech.edu)

**Background**

Paternal Sex Ratio (PSR) is a B chromosome present at low levels in natural populations of the jewel wasp *Nasonia vitripennis*. PSR is transmitted exclusively through sperm and severely biases the wasp sex ratio by transforming diploid embryos, which would normally develop as females, into haploid PSR-carrying males. Other paternally-inherited chromosomes form a so-called paternal chromatin mass (PCM), fail to resolve into individual chromosomes and are eliminated during the first embryonic mitotic division. Molecular mechanisms of PSR-induced paternal genome elimination are poorly understood, although recent study has suggested that altered histone modification state may play a role in that process. Several additional studies have attempted to identify molecular components that participate in the pathway, resulting in the discovery of a number of transcripts, both long and short, that are found exclusively in PSR-carrying populations. However, these studies were not designed to comprehensively catalogue PSR-encoded transcripts, or to characterize the structure of the chromosome. To do that in a thorough and systematic way, the genomic sequence of PSR is required. Previously, the highly repetitive nature of the chromosome, much of which is composed of simple PSR-specific repeats, prevented the assembly of PSR using the now common short read (up to 300 bases long) sequencing technology, such as Illumina. To overcome limitations intrinsic to Illumina data, we employed two recently developed single molecule sequencing platforms, Pacific Biosciences (PacBio) and Oxford Nanopore Technologies (ONT). PacBio monitors the incorporation of fluorescently-labeled nucleotides by a DNA polymerase molecule sequestered in a zero-mode waveguide well (ZMW) in real time, while ONT translates electric signals across a membrane during DNA translocation through a nanopore embedded in the membrane directly into nucleotide sequence. Unlike Illumina, both PacBio and ONT platforms are capable of generating reads that are tens to hundreds of kilobases long making them uniquely suited for assembling repetitive genomes, such as PSR. In this talk, I will discuss the current state of the two long read technologies, their relative strengths and weaknesses as well as challenges of data analysis and integration with other data types. I will then describe how we utilized PacBio and ONT data to generate, for the first time, genomic assembly of the PSR chromosome. Using the new assembly in combination with RNA-seq and small RNA sequencing data, we annotated PSR-encoded transcripts and quantified their expression levels discovering many previously unknown PSR-associated genes. The availability of the annotated genome laid a foundation for functional analyses of PSR-specific genes and led to new insights into the mechanism of PSR function. In addition to the PSR assembly, this work also resulted in a dramatic improvement of the WT genome of *Nasonia vitripennis*, which I will briefly touch upon as well.

### S6-02 Identifying the toxin that causes paternal genome elimination by the B chromosome PSR in *Nasonia vitripennis*

#### Elena Dalla Benetta^1^, Patrick M. Ferree^1^, Omar S. Akbari^2^

##### ^1^W. M. Keck Science Department of Claremont McKenna, Pitzer, and Scripps Colleges, Claremont, California, USA; ^2^Division of Biological Sciences, Section of Cell and Developmental Biology, University of California, San Diego, La Jolla, California, USA

**Correspondence:**  Elena  Dalla  Benetta (dallabenetta.elena@gmail.com)

**Background**

The *Nasonia vitripennis* B chromosome, known as PSR (for Paternal Sex Ratio), causes the complete elimination of the paternal chromatin at each generation, converting female-destined embryos into males, the PSR-transmitting sex. It is thus strictly paternally transmitted through the sperm to new progeny. A compelling question is how PSR causes paternal genome elimination.

**Materials and Methods**

We have employed a combination of different whole genome sequencing platforms (including PacBio, Illumina, and Nanopore MinION) to analyse the fine-scale sequence composition and structure of PSR. We then used this information along with RNA-seq data in order to identify specific B chromosome sequences as toxin candidates.

**Results**

We have found three such sequences that we have tested functionally by parental RNA interference, and have identified one sequence as being involved in genome elimination. Specifically, when this sequence was knocked down by RNAi we observed high numbers of F1 female progeny carrying PSR. Furthermore, cytological imaging showed F1 embryos with both paternal and maternal genomes.

**Conclusions**

These results, although tentative, strongly suggest that PSR induces genome elimination through expression of a unique sequence carried by this B chromosome. We are currently performing transgenic expression experiments to further understand the function of this sequence.

**Acknowledgements**

This work was funded by a United States National Science Foundation CAREER Award (NSF-1451839) to PMF.

